# Expert opinion on the recognition, diagnosis and management of children and adults with Fabry disease: a multidisciplinary Turkey perspective

**DOI:** 10.1186/s13023-022-02215-x

**Published:** 2022-03-02

**Authors:** Fatih Ezgu, Erkan Alpsoy, Zerrin Bicik Bahcebasi, Ozgur Kasapcopur, Melis Palamar, Huseyin Onay, Binnaz Handan Ozdemir, Mehmet Akif Topcuoglu, Omac Tufekcioglu

**Affiliations:** 1 Department of Pediatrics, Division of Pediatric Metabolism and Division of Pediatric Genetics, Gazi University Faculty of Medicine, 06560 Ankara, Turkey; 2 Department of Dermatology, Akdeniz University Faculty of Medicine, Antalya, Turkey; 3 Clinic of Nephrology, Kartal Dr. Lutfu Kirdar Training and Research Hospital, Istanbul, Turkey; 4 Department of Pediatrics, Division of Pediatric Rheumatology, Istanbul University Cerrahpasa Faculty of Medicine, Istanbul, Turkey; 5 Department of Ophthalmology, Ege University Faculty of Medicine, Izmir, Turkey; 6 Department of Medical Genetics, Ege University Faculty of Medicine, Izmir, Turkey; 7 Department of Pathology, Baskent University Faculty of Medicine, Ankara, Turkey; 8 Department of Neurology, Hacettepe University Faculty of Medicine, Ankara, Turkey; 9 University of Health Sciences Department of Cardiology, Ankara City Hospital, Ankara, Turkey

**Keywords:** Fabry disease, Expert panel, Awareness, Screening, Diagnosis, Pathogenic variants, Heterogeneity, Management, Multidisciplinary approach

## Abstract

This consensus statement by a panel of Fabry experts aimed to identify areas of consensus on conceptual, clinical and therapeutic aspects of Fabry disease (FD) and to provide guidance to healthcare providers on best practice in the management of pediatric and adult patients with FD. This consensus statement indicated the clinical heterogeneity of FD as well as a large number of pathogenic variants in the *GLA* gene, emphasizing a need for an individualized approach to patient care. The experts reached consensus on the critical role of a high index of suspicion in symptomatic patients and screening of certain at-risk groups to reveal timely and accurate diagnosis of FD along with an increased awareness of the treating physician about the different kinds of pathogenic variants and their clinical implications. The experts emphasized the crucial role of timely recognition of FD with minimal delay from symptom onset to definite diagnosis in better management of FD patients, given the likelihood of changing the disease’s natural history, improving the patients’ quality of life and the prognosis after enzyme replacement therapy (ERT) administered through a coordinated, multidisciplinary care approach. In this regard, this consensus document is expected to increase awareness among physicians about unique characteristics of FD to assist clinicians in recognizing FD with a well-established clinical suspicion consistent with pathogenic variants and gender-based heterogeneous clinical manifestations of FD and in translating this information into their clinical practice for best practice in the management of patients with FD.

## Background

Fabry disease (FD; OMIM #301500) is an X-linked lysosomal storage disorder caused by deficiency in the lysosomal enzyme α-galactosidase A (α-Gal A), and consequent accumulation of glycosphingolipids, such as globotriaosylceramide (Gb3) and its deacylated derivative globotriaosylsphingosine (lyso-Gb3) within tissues that progressively affect multiple organ systems [1–3].

Fabry Disease is considered as a multi-systemic disorder with progressive neurological, renal, cardiac, ocular and dermatological manifestations [1, 4]. However, even if peculiar signs and symptoms arise in childhood, there is a significant diagnostic delay of up to 20 years from symptom onset [1, 5–8]. This is probably due to the lack of awareness and to the wide spectrum of clinical presentations especially in females [1, 5–8]. Therefore, to recognize the signs and symptoms of FD is closely related to the disease awareness among pediatricians, pediatric metabolic experts, pediatric geneticists, cardiologists, neurologists, dermatologists, nephrologists, trained pathologists, and ophthalmologists [4]. Notably, once the diagnosis is made, it possible to change the disease’s natural history and progression as well as to improve patients’ quality of life (QoL) via treatment [6, 9, 10].

The frequency of inborn metabolic diseases in Turkey is considerably high in general, probably related to the high rate of consanguineous marriages [11]. In accordance with worldwide statistics on an increased awareness of FD among physicians since the disease has become treatable with enzyme replacement therapy (ERT) [12, 13], the heightened awareness about FD is also evident in Turkey, especially after the availability of ERT [14]. Accordingly, in first screening study done in Turkey carried out in a group of male hemodialysis patients using plasma α-Gal A test, authors reported the prevalence of FD to be 0.24% [15]. Afterwards, data from the largest screening study in Turkey that used DBS method for both males and females, along with family screening of the index cases, revealed the prevalence of FD to be 0.17% in all dialysis patients and to be 0.32% in male dialysis patients [16].

However, being a rare disease with a long natural history, FD poses several problems for evidence-based medicine such as difficulty in conducting randomized clinical trials in terms of small sample size, wide clinical spectrum especially in females, unfeasibility of a very long follow-up needed to record end-points, a high probability of enrolling patients with already advanced disease at the recruitment period and ethical problems related to placebo-controlled design given the availability of therapy [17]. Accordingly, along with the considerable variability in the clinical expression of FD and the difficulties in diagnosing the disease, there is a considerable challenge for clinicians in the decision-making process while managing patients with FD due to limited availability of evidence-based solid criteria on therapeutic and prognostic algorithms [6].

Therefore, this review by a panel of Fabry experts aimed to provide guidance to healthcare providers on best practice in recognition, diagnosis, and management of pediatric and adult FD patients through a practical and implementable document with a comprehensive framework addressing the conceptual, clinical, and therapeutic aspects of FD mainly focusing on the general approach in Turkey.

## Methods

The present panel of FD experts from ten different specialties involved in the management of patients with FD including nephrology, cardiology, neurology, dermatology, ophthalmology, pediatric metabolic diseases, pediatric genetics, pediatric rheumatology, medical genetics, and pathology convened in Istanbul, Turkey to develop a consensus opinion on the conceptual, clinical and therapeutic aspects of FD from the Turkey’s perspective. The participating Fabry experts with at least 15 years of experience in dealing with FD in different provinces of the main geographical regions of Turkey were informed about the study via e-mail by the sponsor and then participated in the consecutive meetings to achieve the proposed consensus. The panel critically analyzed recommendations from existing guidelines and data from systematic reviews, meta-analyses and literature review of articles published on FD in pediatric and adult populations and agreed on a series of statements supported by scientific evidence and expert clinical opinion to assist healthcare providers on best practice in recognition, diagnosis, and management of FD. The development of the consensus document was also guided by a number of key questions regarding specialty-based diagnostic criteria, diagnostic challenges, high risk individuals, biomarkers as well as optimal management and follow up of patients. The proposed consensus planned to provide a practical and implementable guidance document addressing FD in terms of (a) definition and epidemiology, (b) phenotypes and clinical manifestations according to patient age (early, late) and specific organ involvement (renal, cardiac, nervous system, ocular, dermatological), (c) diagnosis and biomarkers, (d) clinical suspicion, screening and genetic counselling, (e) treatment (ERT, adjuvant therapy), and (f) role of multidisciplinary approach in evaluation and management of the disease.

## Phenotypes and clinical manifestations

FD (Online Mendelian Inheritance in Man [OMIM] #301500), is a progressive X-linked lysosomal storage disorder, caused by deficiency of lysosomal enzyme α-Gal A (Enzyme Commission [EC] number 3.2.1.22) due to pathogenic variants in the encoding *GLA* gene (OMIM #300644; HGNC 4296) [1, 2, 4, 18]. This results in loss of function of the enzyme and deposition of glycosphingolipids, such as globotriaosylceramide (Gb3) and its deacylated derivative globotriaosylsphingosine (lyso-Gb3), within lysosomes in virtually all cell types including capillary endothelial cells, renal cells (podocytes, tubular cells, glomerular endothelial, mesangial, and interstitial cells), cardiac cells (cardiomyocytes and fibroblasts) and nerve cells [1, 2, 4, 19].

The primary disease process, through this continuous deposition, starts as early as during the fetal stage of development, whereas unlike to many other lysosomal storage diseases, most patients remain clinically asymptomatic during the very first years of life until occurrence of first symptoms at ages of 3 to 10 years, and generally a few years later in girls than in boys [1, 20–22].

With advancing age, significant lysosomal and cellular dysfunction probably trigger a cascade of events (i.e. cellular death, inflammation, small vessel injury, oxidative stress and tissue ischemia) and results in development of a multisystem disorder via progressive damage to the vascular endothelia, particularly of small vessels, affecting multiple vital organ systems including the heart, nervous system, and kidneys [1, 4, 23]. Accordingly, affected patients are at high risk of developing fibrotic cardiac disease resulting in rhythm and conduction disturbances, progressive hypertrophic cardiomyopathy (HCM), progressive proteinuric kidney disease, a small-fiber neuropathy and mostly ischemic cerebrovascular stroke [23–26].

The estimated incidence of FD in the general population is 1 in 117,000 and 1 in 40,000 males [4, 10, 27], while this has been considered a significant underestimation given the under-recognition of symptoms and delayed or missed diagnoses [4, 10]. Accordingly, newborn screening studies revealed overall incidence estimates of 1 in ∼1250–3100 males [28, 29], although a prevalence of around 1 in 8000 is also considered more likely when benign variants of the *GLA* gene have been excluded [1, 4].

The natural course of FD indicates that the mean survival of patients is about 55 years for males and about 70 years for females with an ~ 17-year and 5-year shortened life span, respectively [30, 31]. This is important given that once a diagnosis is made, the disease can be effectively and safely controlled with treatment [9, 10, 32].

FD has two major phenotypes including “classic” and “late onset” subtypes [8, 13, 33]. The classic (typical) phenotype is characterized by little or no residual α-Gal A enzyme activity and onset with the typical early symptoms (i.e. acroparesthesias, angiokeratoma, hypohidrosis and/or a characteristic corneal dystrophy) during childhood/adolescence, particularly in males. “Non-classical” form is also referred as “later-onset” form and includes “cardiac” or “renal” variants with no/minimal involvement of other organs. The clinical manifestations in heterozygous females range from asymptomatic throughout a normal life span to as severe as affected males. Variation in clinical manifestations is attributed to X-chromosome inactivation pattern. With advancing age, the progressive deposition of glycosphingolipids lead to progressive multi-system involvement that results in renal failure, HCM, and/or cerebrovascular disease [2, 13, 24, 25, 33]. The late onset (atypical) phenotype is typically less severe with a significant residual α-Gal A activity in males, who remain clinically asymptomatic until the 4–7th decades of life, when they develop progressive organ damage and present with a cardiomyopathy or end-stage renal disease (ESRD) and/or cerebrovascular insult [1, 8, 12, 13, 34, 35].

In fact, the age of onset of symptoms, the extent of organ involvement, and prognosis of FD depend both on the underlying degree of α-Gal A deficiency and the gender of the patient [2, 36, 37]. Males with higher residual enzyme activity tend to have later onset disease with predominantly single organ forms, while females tend to have milder and slowly progressing disease phenotypes as well as a wider spectrum of disease severity (ranging from asymptomatic to severely affected phenotype) than males [2, 5, 36, 37].

This variation in the phenotype and disease course among female patients, that ranges from being asymptomatic throughout a normal life span to as severe as many affected males, has been attributed to the severity of the pathogenic variants and X-chromosome inactivation [5, 38, 39]. More severely affected females are more likely to have the X-chromosome with the *GLA* wild-type gene inactivated and the X-chromosome carrying the pathogenic variant remaining expressed in the affected organs [8, 22, 25, 33].

Besides these factors, a recent study from Turkey suggested that co-existent factors (such as increased lipoprotein (a), homocysteine, total and low density cholesterol and antithrombin 3 levels; prothrombin p.G20210A and factor V Leiden pathogenic variants) or diseases (such as rheumatologic diseases or celiac disease) could significantly modify the phenotype in both females and males should be considered as a part of initial work-up [14].

FD is further characterized by a large number of pathogenic variants in the *GLA* gene, including variants associated with the classic phenotype, later onset phenotype and benign variants and variants of uncertain significance (VUS) [1, 2, 40, 41].

## Clinical manifestations specific to age

### Early signs and symptoms: FD in pediatric and adolescent age

In classic FD, the first symptoms that may emerge in childhood or early adolescence are skin abnormalities (angiokeratomas), corneal deposits (cornea verticillata), microalbuminuria and/or proteinuria, and symptoms related to autonomic nervous system involvement including acroparesthesia (chronic neuropathic pain), diffuse episodic pain crises (Fabry crises), and sweating abnormalities (anhidrosis or hypohidrosis) [1, 39, 42]. Tinnitus may be an early symptom and hearing loss has been reported in children, while chronic fatigue and difficulty gaining weight may also frequently occur, particularly during adolescence [1]. Early signs of cardiac (shortened PR interval, arrhythmias, chronotropic incompetence, aortic dilation at the Valsalva sinuses and mild valvular insufficiency) and cerebrovascular abnormalities (cerebral small vessel involvement) may also be detectable during adolescence in both genders (Table 1) [43, 44].

**Table 1:** Signs and symptoms of Fabry disease [1, 39, 42–46]

Organ system	Clinical manifestations (signs/symptoms)	
	Pediatric	Adult
Peripheral neuropathy	Neuropathic pain (Fabry crises)	Present or past experience of neuropathic pain
	Hypohidrosis, heat intolerance	Hypohidrosis, heat intolerance
	Hearing loss, vertigo	Hearing loss, vertigo
	Nausea, vomiting, diarrhea	Nausea, vomiting, diarrhea
	Abdominal pain	Abdominal pain
	Postprandial bloating and pain, early satiety	Postprandial bloating and pain, early satiety
	Difficulty gaining weight	Difficulty gaining weight
Cerebrovascular involvement	Cerebral microvascular ischemic involvement	Significant white matter lesions
		Transient ischemic attacks
		Ischemic strokes
		Neuropsychiatric phenotype
Renal	Microalbuminuria, proteinuria	Albuminuria overt proteinuria
		Progressive chronic kidney disease
		Kidney failure
		End-stage renal disease necessitating renal replacement treatments
Cardiac	Impaired heart rate variability	Left ventricular hypertrophy
	Arrhythmias	Hypertrophic cardiomyopathy
	ECG abnormalities (shortened PR interval)	Cardiac arrhythmias
	Mild valvular insufficiency	Valvular disease
	Aortic dilatation at Valsalva sinuses	
Dermatological	Angiokeratomas	Angiokeratomas
	Sweating abnormalities (hypohidrosis)	Linear telangiectasia
		Lymphedema
		Hypotrichosis
Ocular	Corneal deposits (cornea verticillata) and lenticular opacities	Corneal deposits (cornea verticillata) and lenticular opacities
	Vasculopathy (conjunctiva, retina)	Vasculopathy (conjunctiva, retina)

Importantly, these symptoms, albeit not accompanied with major organ dysfunction, can be significant cause of morbidity by negatively affecting physical, school and social performances of children [45].

### Late/advanced signs and symptoms: FD in adulthood

In adulthood, with disease progression, patients are at significant risk of ESRD necessitating early dialysis and other renal replacement treatments and the development of serious cardiovascular [i.e. left ventricular hypertrophy (LVH), HCM, cardiac arrhythmias, valvular disease] and cerebrovascular complications [i.e. cerebral white matter lesions (CWMLs), transient ischemic attacks (TIAs), ischemic strokes] that can cause premature death [1, 39, 42]. In addition, many adults continue to suffer from debilitating pain and some adult patients display a unique neuropsychiatric phenotype, characterized by subtle movement impairment and depression, resulting in reduced QoL (Table 1) [1, 42, 46].

Fabry Disease can have overlapping signs and symptoms with other disorders such as Familial Mediterranean Fever (FMF), juvenile systemic lupus erythematosus and celiac disease which are frequently seen in Turkey. Fabry Disease should also be considered for patients with a preliminary/confirmed diagnosis of such disorders as the real etiologic/co-existent disease [47–49].

## Clinical manifestations specific to organ involvement

### Renal involvement

Fabry nephropathy is related to progressive decrease in renal function with onset of deposition of Gb3 in almost all renal cell types (i.e. vascular endothelial cells, vascular smooth muscle cells, mesangial cells, interstitial cells, podocytes, and distal tubular epithelial cells) as early as during fetal development [8, 50]. Increase in microalbuminuria and proteinuria are the initial manifestations of renal impairment that occur as early as 10 years of age or earlier, while a decline in glomerular filtration rate (GFR) is seen starting from the adolescence in classic patients. [1, 51].

With progressive chronic kidney disease (CKD) including albuminuria and overt proteinuria in the second decade of life [52–54], renal pathology increases in severity and chronic renal insufficiency and ESRD develop ultimately in the 3–5th decades and 4–5th decades of life, respectively [1, 8, 55, 56].

The dialysis is initiated due to ESRD among male patients with FD in the 4–5th decades of life [57] and kidney dysfunction is less prevalent and less severe among female heterozygous patients, while some cases of ESRD among heterozygous females have also been reported [58].

#### The consensus statement on Fabry-specific renal involvement

**Fig. 1:**
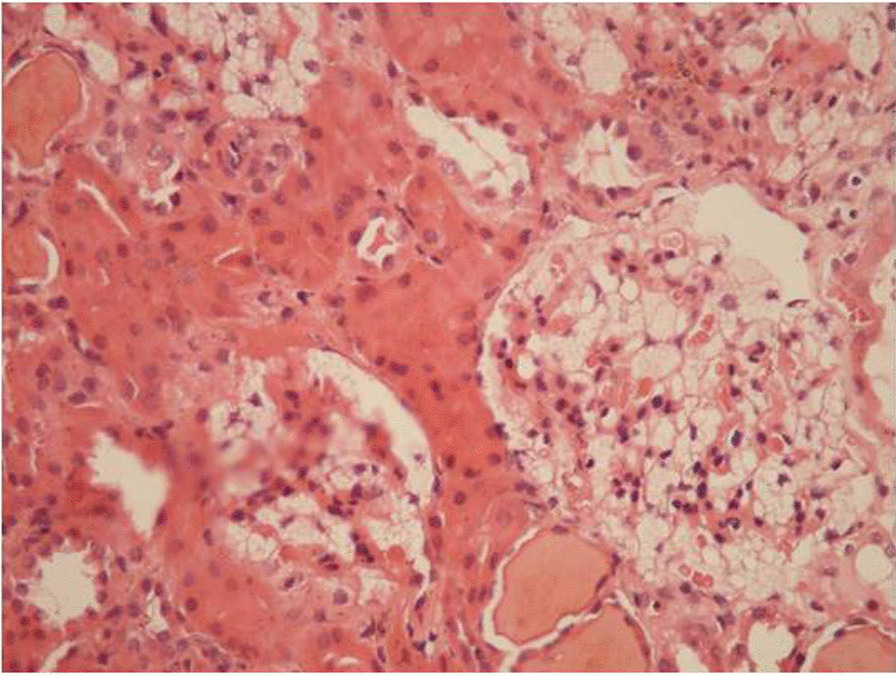
Pathologic findings of renal involvement of Fabry disease in kidney biopsy, light microscopy (Hematoxylin Eosin staining, X400 magnified)

- Laboratory investigations of kidney function that should be carried out in every patient include serum creatinine, cystatin C, estimated GFR (e-GFR), urinary protein, albumin and/or microalbumin excretion and urinary sodium excretion [59]. Assessment of proteinuria and GFR can be used for the staging of CKD, while urinary protein excretion is strongly and independently associated with renal disease progression in FD, regardless of the gender [1, 60, 61].
- Light microscopy in biopsy specimens from kidney shows the accumulation of sphingolipids as PAS-positive Sudan-positive intra-lysosomal inclusions viewed under polarized light [62]. However, this does not usually contribute a great deal to diagnosis given that exposure of biopsy material to lipid-dissolving materials in preparation for light microscopic evaluation results in non-specific findings (foamy podocytes) likely to be observed in any disease associated with nephrotic range proteinuria (Fig. 1).
- Electron microscopy*-*based ultrastructural studies of kidney biopsies can reveal lysosomal storage in a variety of kidney cellular types, based on identification of the inclusions as whorled layers of alternating dense and pale material (‘zebra bodies’ or myelin figures) [1].
- Importantly, when electron microscopy is not available, preparation of semi-thin (1 micron) sections with osmium tetroxide and glutaraldehyde fixation and toluidine blue or Masson’s trichrome staining is considered to allow diagnosis with detection of zebra bodies when viewed under polarized light without using electron microscopy [1].
- Given that potentially irreversible changes to glomeruli, interstitial tubules and vascular structures can be observed in renal biopsy specimens even before the first appearance of microalbuminuria in a child, the histological changes are considered to be an early indicator of renal damage and diagnostic as well as prognostic indicators in FD [63].
- Histological studies should not routinely be performed for the diagnosis of FD, while a biopsy of the affected organ for a histological study with electron microscopy is necessary in cases of suspected FD having indefinite enzyme and/or genetic test (especially VUS) results [6]. In addition it should also be noted that, acquired metabolic disorders, such as the one induced by chloroquine therapy, may result in storage of ultrastructurally similar inclusions in many of the same cells as FD, leading to erroneous interpretation [64].

### Cardiac involvement

Cardiac manifestations occur in 40–60% of FD patients with similar spectrum in the classic and the late onset cardiac phenotype including LVH, conduction abnormalities, bradycardia and chronotropic incompetence, supraventricular and ventricular tachyarrhythmia, myocardial fibrosis, valve disease, aortic dilation at Valsalva sinuses and microvascular dysfunction [65–67].

Right ventricular hypertrophy with diastolic dysfunction is also considered to be common in FD, being responsible for clinical features such as reduced exercise capacity, organomegaly and lymphedema in patients with preserved left ventricle function [68]. According to data from the Fabry Registry, heart failure occurs in 3.5% in men and 2.3% in women with FD [66], while myocardial fibrosis is a marker for poor prognosis as associated with increased risk of malignant ventricular arrhythmias and sudden cardiac death [69–71].

In FD, men aged > 30 years and women aged > 40 years most often present with LVH unexplained by abnormal cardiac loading conditions (i.e. hypertension or aortic stenosis) and usually concentric and non-obstructive [1, 4]. However, sometimes the picture mimics sarcomeric HCM, particularly when isolated, as in the cardiac or late-onset variant of the disease, and cardiologists should therefore be aware of the cardiac variant of FD in case of HCM [1, 4]. Notably, in HCM cohorts, up to 1–3% of patients have been diagnosed with FD, while the prevalence of HCM resulting from sarcomeric gene pathogenic variants is reported in 1/500–1/200 of the general population, and at least 10% of HCM among adults is estimated to have non-sarcomeric origins, arising rather from amyloidosis, FD or mitochondrial diseases, necessitating specific treatments [4, 72].

#### The consensus statement on Fabry-specific cardiac involvement

**Fig. 2:**
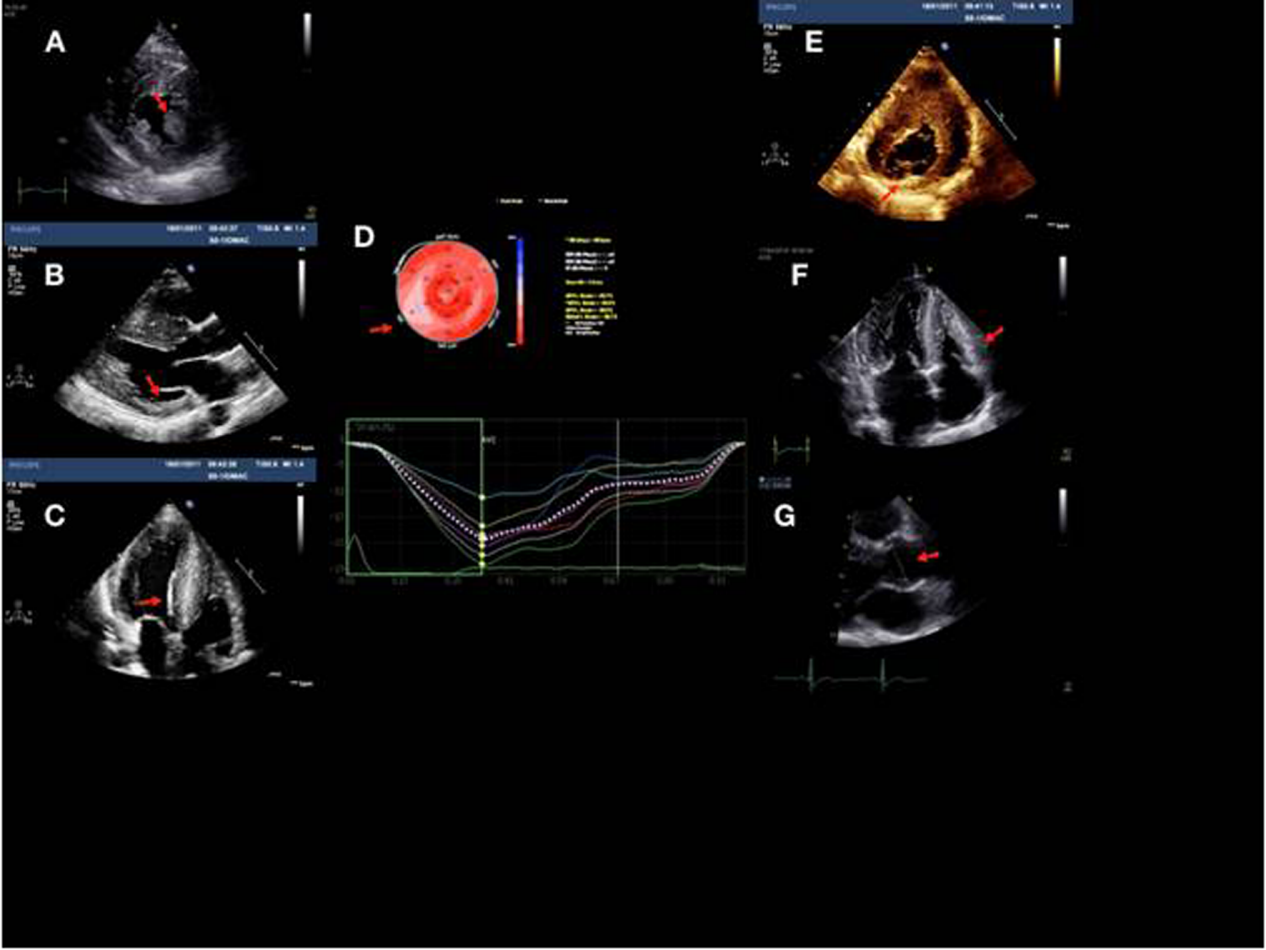
Echocardiography findings in a patient with Fabry disease. **A** Hypertrophic papillary muscle, **B** thinning in inferior and posterior basal walls of the left ventricle in an advanced case, **C** “Binary sign”, **D** typical “Strain Bull’s Eye” in FD, segmenter longitunal strain reduction in infero-basal wall, **E** increase echogenicity in areas with advanced myocardial fibrosis, **F** right ventricular hypertrophy and valvular thickening, **G** Aortic dilation at Valsalva sinuses

**Fig. 3:**
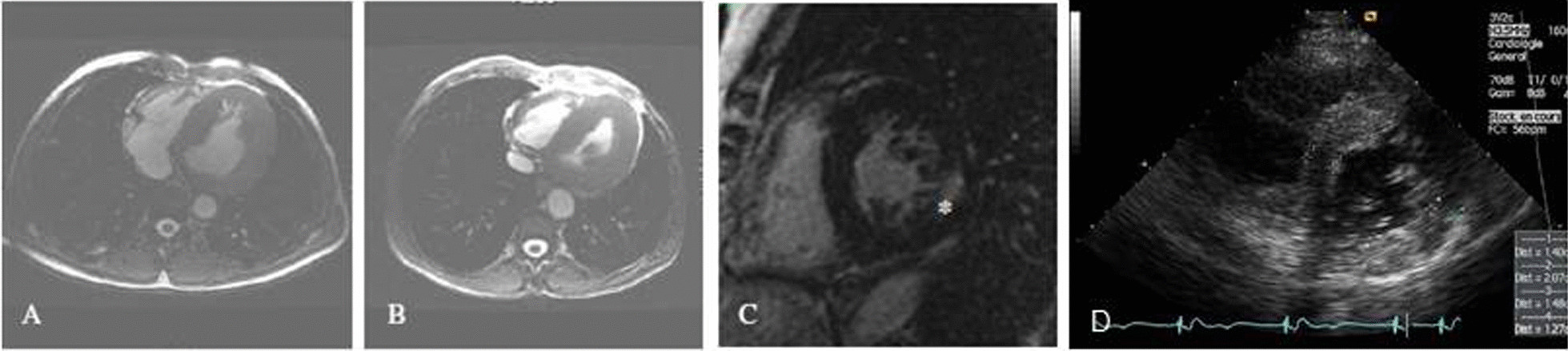
Cardiac MRI for the assessment of left ventricular hypertrophy and fibrosis. **A** Left ventricular hypertrophy in a 51-year-old male patient with cerebrovascular involvement and end stage renal disease (dialysis). **B** Hypertrophic cardiomyopathy in a 56-year-old male patient with arrythmya, leukoareiosis and kidney transplant. **C** Late enhancement after gadolinium in a 63-years-old female patient with end stage renal disease (dialysis). **D** Echocardiography: parasternal short axis showing left ventricular hypertrophy. Adapted from Germain Orphanet Journal of Rare diseases 2010, 5:30

- The initial cardiac evaluation should include resting 12-derivation ECG, long-duration electrocardiogram recordings, echocardiography, and late gadolinium and T1 mapping magnetic resonance imaging (MRI) [36]. Abnormalities of a non-hypertrophied inferolateral wall at the base of the left ventricle and low native T1 signal on MRI are evocative [4].
- Diagnosis of LVH is usually made initially by echocardiography to assess the extent and pattern of LVH and evaluation of cardiac dysfunction, which typically combines concentric thickening without left ventricular obstruction and normal LVEF (Fig. 2) [4, 8].
- However, asymmetric septal or apical hypertrophy has also been described, along with sub-aortic obstruction, which may mimic the phenotypical and clinical features of sarcomeric HCM [72–74]. In such cases T1-mapping MRI is useful, given that a low native T1 is specific to FD cardiomyopathy, while very uncommon in HCM due to sarcomere gene pathogenic variants, amyloidosis, or hypertension [75]. Although low native T1 can be also observed in haemochromatosis, this can be eliminated based on low T2* values [74]. Low nativeT1 values of the myocardium secondary to sphingolipid storage are noted in 40% of patients with FD without LVH and in > 90% of patients with FD with LVH, indicating T1 mapping a useful test for early detection of cardiac involvement, even in the absence of LVH [4, 75, 76].
- Indeed, cardiac MRI is becoming increasingly important for the diagnosis of cardiac involvement in FD, since it is able to identify patients with fibrosis as well as LVH more precisely and T1-mapping MRI and strain echocardiography might be more sensitive for the early detection of the cardiac manifestations of FD (Fig. 3) [72–74].
- Cardiac MRI also offers higher definition of ventricular structures and visualization of the extent of scarring and fibrosis and more reproducible quantification of LVH for serial within-patient assessments, which is important given that in female patients, cardiac fibrosis may be present before LVH (Fig. 3) [36, 77].
- As MRI is widely available in Turkey, an effort should be made to establish referral centers in several regions specialized for the MRI based cardiac evaluation of patients.

### Nervous system involvement

The early peripheral neuropathic hallmarks of FD are frequently followed by cerebrovascular complications and autonomic dysfunction in adulthood [1].

#### Small-fibre peripheral neuropathy

The first neurological symptoms of FD occur due to small-fibre peripheral neuropathy and involve a spectrum of manifestation including peripheral neuropathic pain, deficits of thermal sensation and of physiologic pain perception, impaired sweating, GI dysmotility, and other sensory deficits such as hearing loss [36]. In Fabry neuropathy, both myelinated and non-myelinated nerve fibers are reduced. Gb3 accumulation occurs mainly in the dorsal root ganglion neurons and Schwann cell. However, there is also accumulation in the perineurium, vascular smooth muscle cells, fibroblasts, and endothelial cells. As a main mechanism, a dying back neuropathy is thought to be caused by neuronal damage in the dorsal root ganglion. This explains the intraepidermal nerve fiber density reduction well. Another mechanism involved is chronic nerve ischemia [36].

#### Neuropathic pain (Fabry crises)

Fabry-related neuropathic pain due to small-fibre neuropathy is often the earliest manifestation for children with classical FD (60–80%) that can occur as early as 3 years of age or less [78], usually occurring at an earlier age in boys than in girls [79, 80]. The pattern of neuropathic pain in FD is considered to involve evoked pain (allodynia or hyperalgesia), pain attacks, permanent (chronic) pain and pain crises along with likelihood of experiencing one or more types concomitantly or a change in the pattern over time [36, 81].

#### The consensus statement on Fabry crises

The most common presentation of neuropathic pain among FD patients is Fabry pain crises characterized by agonizing burning pain originating in the extremities and radiating inwards to the limbs and other parts of the body, and are often precipitated by rising body temperature due to exercise, fever, or warm ambient environments [1, 39, 58, 82, 83]. An elevated erythrocyte sedimentation rate is commonly noted when the crises are triggered or accompanied by fever, while patients also have a greatly diminished QoL as a result of their pain [1]. In children, other possible causes of small-fiber neuropathy pain that must be ruled out are autoimmune diseases that were usually organ specific and often autoantibody associated including autoimmune thyroiditis, Henoch–Schönlein purpura, brachial plexitis, type 1 diabetes, post-viral arthritis, immune thrombocytopenic purpura, Crohn's disease, autoimmune trochleitis, Hashimoto's encephalopathy, Sjögren's spectrum disorders, rheumatoid arthritis, rheumatic fever, systemic lupus erythematous, and Raynaud's disease as well as ‘growing pains’ (a frequent misdiagnosis in children with FD) [1, 47, 84, 85].

Pain may wane in adulthood and it is very important to search for a medical history of Fabry crises in childhood during the first examination of a newly diagnosed adult patient [84].

The prevalence of pain among adults with FD may be up to 80%^55^ and in adults with chronic pain, celiac disease and multiple sclerosis are the most often-cited differential diagnoses, particularly in females. [1, 85, 86].

#### Hypohidrosis

Absence of sweating (anhidrosis) or a decreased ability to sweat (hypohidrosis) with decreased skin impedance has been reported to occur in 53% of males and 28% of females with FD due to due to a dysfunction of sympathetic sudomotor fibres [87–89], and considered a significant problem leading to hyperthermia, poor exercise tolerance, and altered fever manifestation [88, 90]. Peripheral sweat production can be measured with quantitative sudomotor axon reflex tests (QSART); sympathetic skin response and heart rate variability are considered less reliable tests [91].

#### Hearing loss and vertigo

Auditory and vestibular abnormalities are frequent deficits observed in FD, resulting in a range of symptoms, such as symptomatic (18–55%) hearing loss (more commonly in classic vs. late onset phenotype) as well as tinnitus and vertigo [92–94]. It is important to know the cause of hearing impairment prior to treatment initiation; audiometry testing and neurological investigations should therefore be carried out at diagnosis and at regular intervals following diagnosis [1, 36, 92]. Recently in a study from Turkey Reflex Decay Test was proposed to be an early indicator of hearing loss due to Fabry Disease [95].

#### Gastrointestinal dysfunction

Gastrointestinal (GI) symptoms are common, but under-appreciated, manifestation of FD, as some of the earliest and most commonly reported symptoms that usually remain present also during adulthood [1]. Including abdominal pain, bloating, diarrhea, constipation, nausea, and vomiting as well as failure to gain weight [36], GI dysfunction has been associated with deposition of Gb3 in the autonomic ganglia of the bowel and mesenteric blood vessels [96, 97]. In evaluation, use of a validated GI symptom rating scale is important to enable the progression of GI symptoms to be monitored, while other techniques such as endoscopy, scintigraphy, video capsule endoscopy, and intestinal biopsy can also be used to investigate GI symptoms. [36, 97]. Diarrhea-predominant irritable bowel syndrome (IBS) is a differential diagnosis [96].

#### Cerebrovascular involvement

Cerebrovascular manifestations in FD patients range from mild to severe and include headache, vertigo/dizziness, transient ischemic attacks, recurrent ischemic strokes, and vascular dementia [8, 98]. The overall prevalence of ischemic stroke or transient ischemic attack in patients with FD is considered to be 6.9% for males and 4.3% for females [99], while prevalence of FD in young patients with cryptogenic ischemic strokes (aged 18–55 years) is 0.3–1% [100, 101]. The 4th decade of life seems to be critical for the progression of white matter hyper-intensities, while no strong correlations have been noted between white matter hyper-intensities and other typical FD phenotype manifestations such as renal or cardiac involvement, pain scores, enzyme activity or lyso-Gb3 levels [102, 103].

The prevalence of basilar artery dolichoectasia (extensive remodeling of the vessel with the morphological changes regarding the diameter, the elongation, and the tortuosity) among the FD population is not well defined, but recent data showed that it could be an early marker of cerebrovascular disease [36, 104].

#### The consensus statement on Fabry-specific cerebrovascular involvement

**Fig. 4:**
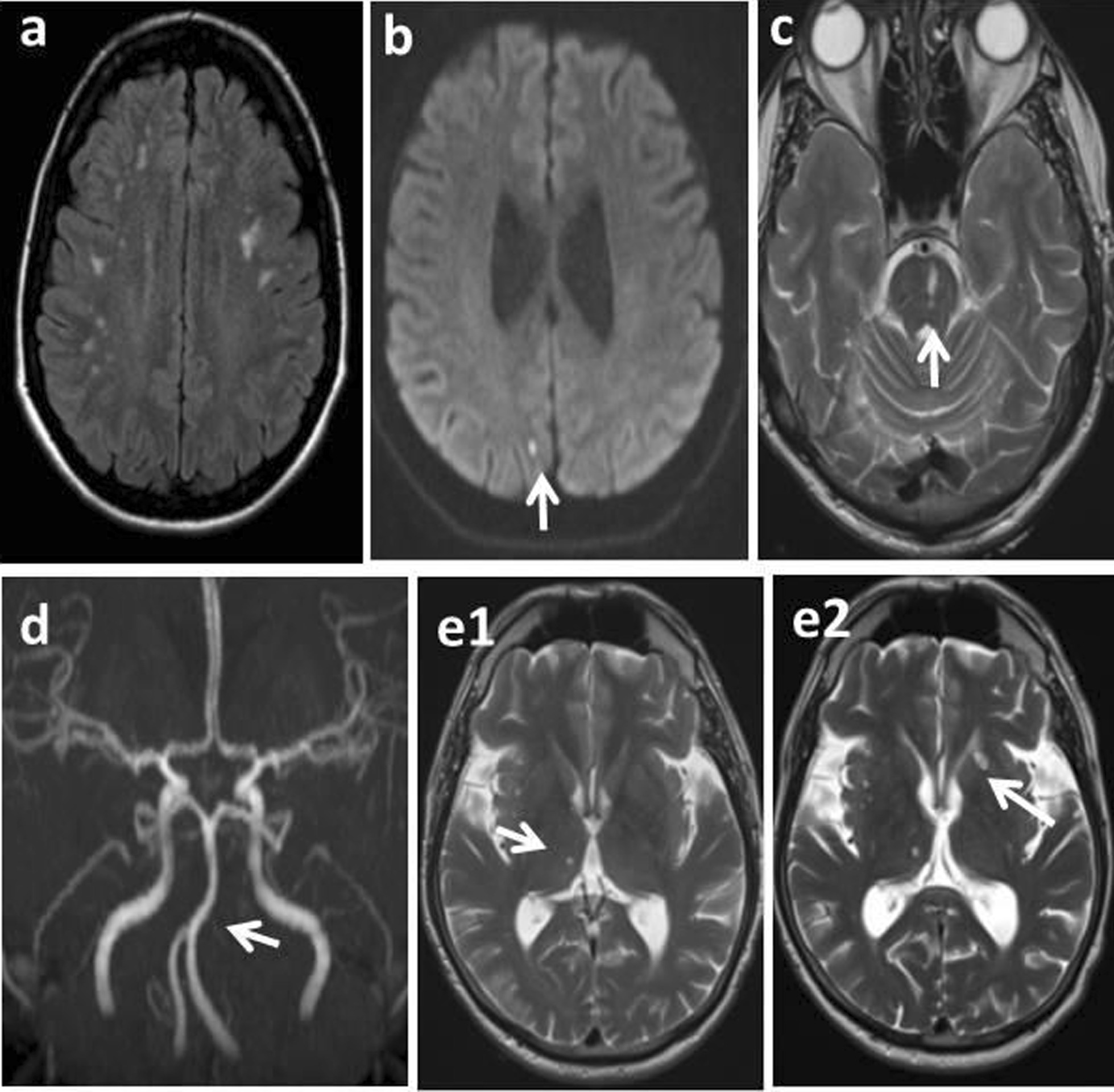
Neurological involvement in Fabry disease. Spectrum of brain MRI findings in a 51-year-old male patient with Fabry disease: **a** White matter hyperintense lesions in FLAIR sequence, **b** acute small-junctional infarct (arrow) in DWI, **c** Chronic left midpons perforating infarcts (lacune type, arrow) in T2 weighted images, **d** Basilary dolichoarteriopathy (arrow), e1–2) Accumulation of small basal perforating infarcts: **e1**, right thalamic lesion, **e2**, emergence of left caudate lesion in 1 year interval

- Imaging modalities that can be used to explore cerebrovascular involvement in Fabry patients include brain CT and CT angiography, MRI and MR angiography in the routine settings, and trans-cranial Doppler (TCD), proton MR spectroscopy (MRS), positron emission tomography (PET) and diffusion tensor imaging in selected cases [1].
- Neuro-radiological findings related to cerebrovascular manifestations of FD include chronic white matter hyper-intensities, the basilar artery dolichoectasia (diameter greater than 4.5 mm at the midbasilary region), microbleeds, cortical and deep small infarcts (Fig. 4) [1, 98, 105–107].
- Given that all of these neurological manifestations may also accompany other disorders such as hypertension, hyperlipidemia or coagulopathies [8], the younger age of presentation seems to be distinctively characteristic of FD. Morphology and topography of cerebral ischemic lesions and infarcts in FD have no diagnostic features. Therefore, Fabry should always be considered in all cases of cryptogenic young stroke (age < 50). However, if young people have thrombotic small vessel disease, Fabry should be among the first priority diagnoses.
- In addition, pulvinar sign (hyperintense appearance of thalamic pulvinar nuclei on T1-weighted images) reflecting the presence of micromineralization has also been considered a highly specific key imaging sign indicative for FD in the presence of other diagnostic clues, being more frequent in male patients with cardiomyopathy and severe kidney involvement. Albeit highly specific, pulvinar sign is a rare finding, at the level of one-fifth of males and case-report among females (Fig. 4) [108–110].
- FD can be misdiagnosed as multiple sclerosis because patients with either disease can present with acute attacks of neuropathic pain in the limbs, especially under conditions of stress, heat, or fatigue and white matter lesions (CMWL) on MRI [86, 111]. Although the pattern of CWML in FD demonstrates a distribution frequently referred to as “vascular leukodystrophy”, variability in appearance due to aging and the temporal lesion load can confound the differential diagnosis [112]. Nevertheless, spinal cord involvement with characteristic neuroradiological findings, when present, is an additional powerful diagnostic element in MS [113]. Usually a careful neuroradiological analysis should be able to distinguish between white matter lesions that are highly suggestive of inflammatory events and MS, from those that are more typical of vasculopathy and FD [111, 113].

### Ocular involvement

Ophthalmological manifestations in FD occur as early as the first decade of life and include cornea verticillata, increased vessel tortuosity, Fabry cataracts and symptomatic conjunctival telengiectasias and aneurysm like formations [114, 115].

**Fig. 5:**
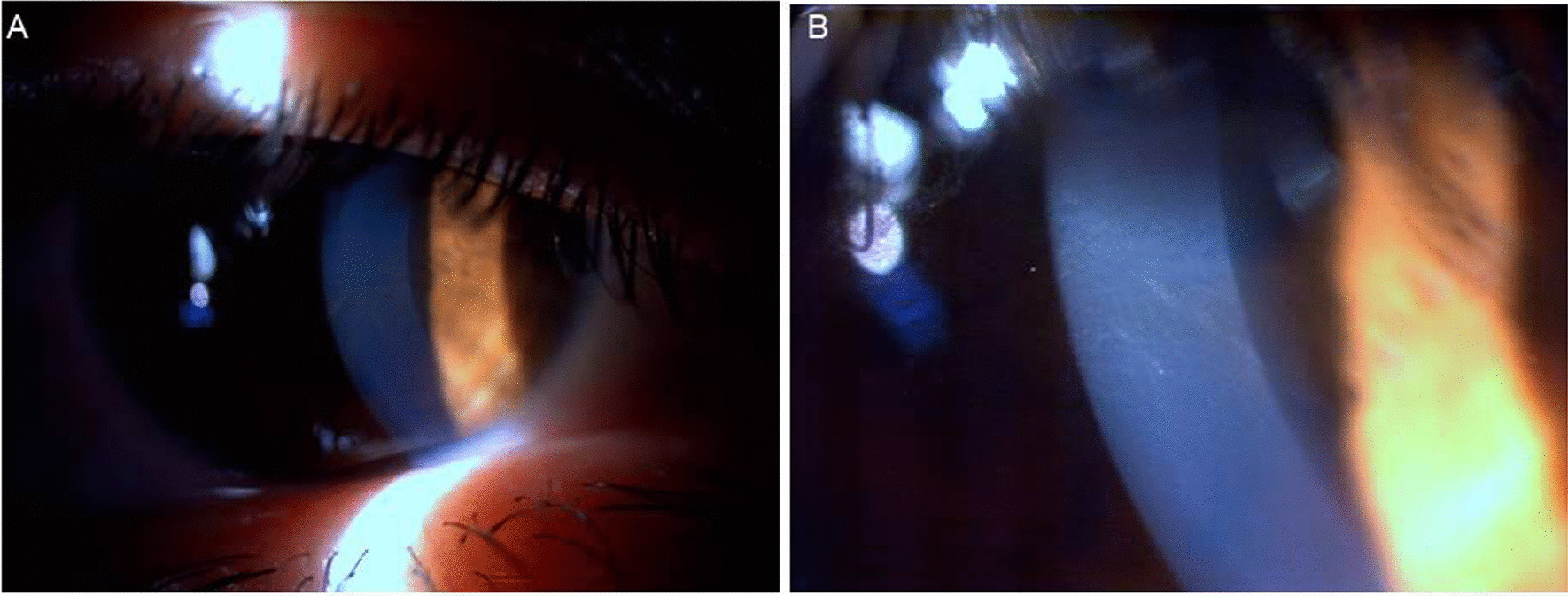
**A** Biomicroscopic appearance of “cornea verticillata” in the right eye. **B** A higher magnification of the same patient demonstrating the whorl-like pattern of cornea verticillata

Cornea verticillata (whorl-like, linear pigmentation in the inferior part of the cornea visible by slit-lamp microscopy) manifests as almost pathognomonic corneal deposits considered among the most common and early of ocular signs, occurring in majority of classical males [114–117]. The observation of typical cornea verticillata is highly predictive for the diagnosis of classic FD, while amiodarone or chloroquine can also produce similar ophthalmological signs (Fig. 5) [114, 117, 118]. Fabry cataract is a posterior capsular cataract with visible whitish spoke-like deposits of granular material and considered a pathognomonic ocular sign of FD [114, 119, 120].

**Fig. 6:**
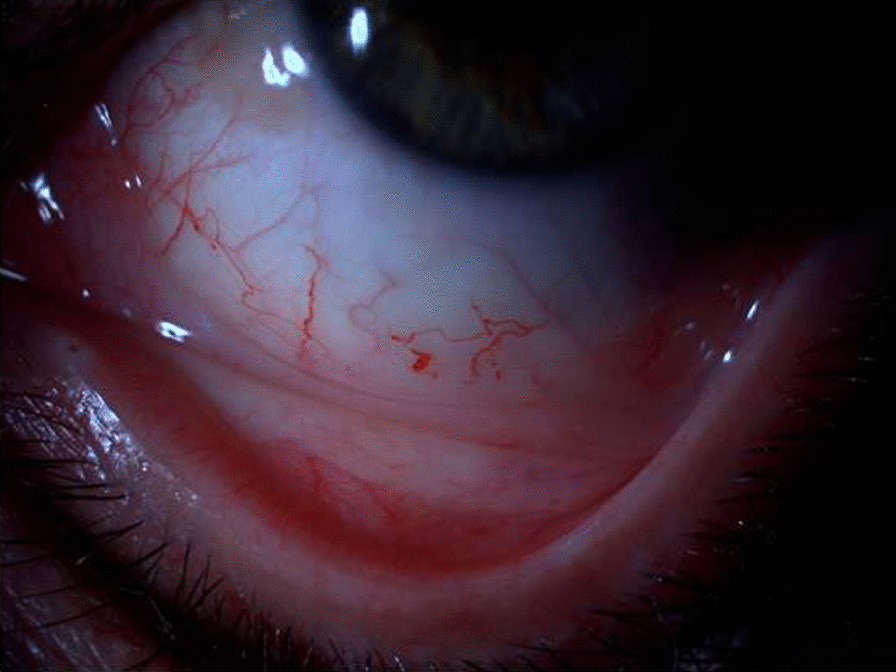
Vascular tortuosity increase, telengiectasias, and aneurysmal changes of the conjunctival vessels of the left eye of a Fabry patient

**Fig. 7:**
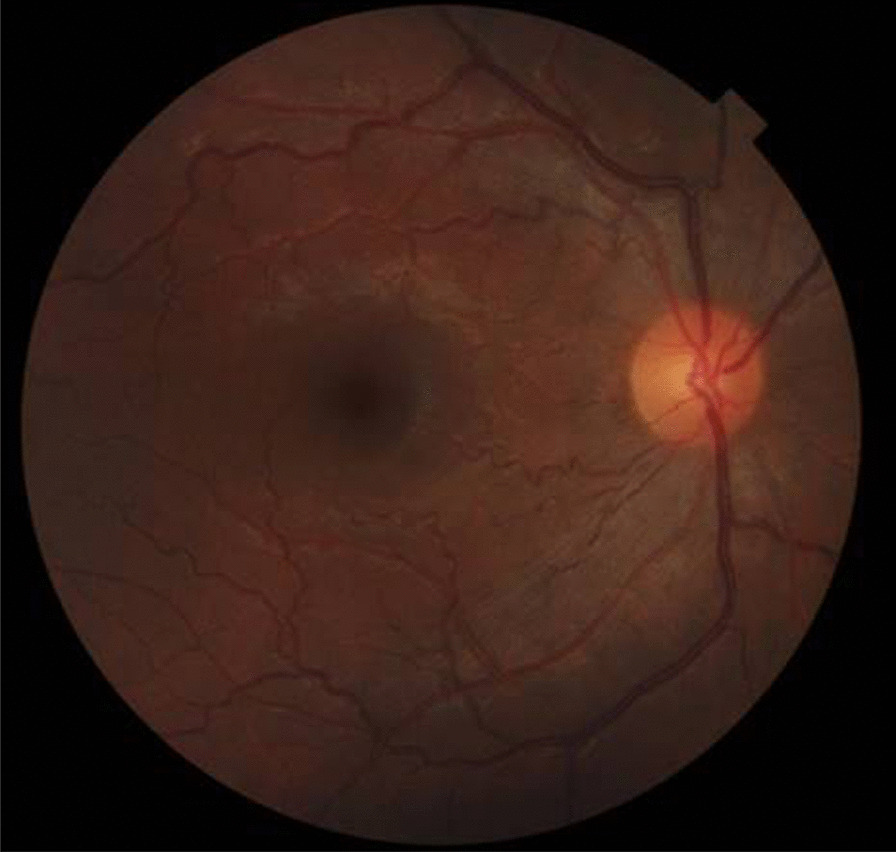
Fundus examination of a Fabry patient showing increased retinal vascular tortuosity in the right eye

Conjunctival telengiectasias, dilatation and tortuosity, and aneurysm formation of the conjunctival vessels are also considered as relatively common signs in patients with classic FD (Fig. 6). Dry eye syndrome is also one of the frequent symptoms in FB and should be monitored and treated if needed [114]. Mild to marked increased tortuosity of the retinal vessels are also observed in patients with FD (Fig. 7) [116, 120].

Cilioretinal artery occlusion and anterior ischemic optic neuropathy were also reported in a female patient with Fabry Disease from Turkey [121].

#### The consensus statement on Fabry-specific ocular involvement

- Ophthalmologic examination provides a unique and important opportunity to diagnose FD. Any patient with corneal haze, cornea verticillata, or Fabry cataract, especially in combination with retinal vascular tortuosity, conjunctival vascular telangiectasia, or lenticular opacities, should undergo a thorough review of symptoms and family history. Slit-lamp examination will be needed to reveal cornea verticillata [115].
- Ophthalmic findings in FD are more common in classical vs. late onset variant of disease [122]. Given that cornea verticillata is the most frequent eye finding in FD that can be easily detected by slit-lamp bio-microscopy with minimal inter- and intra-observer variability and independent of the influence of aging or environmental factors [115], potential use of cornea verticillata has been emphasized as a biomarker for FD [114, 115].
- Fabry cataract is usually regarded to be pathognomonic, whereas mannosidosis might cause the similar lens opacity and increased ocular vessel tortuosity has also been reported in healthy individuals as well as in fucosidosis [114, 115, 122–124].
- In addition, light microscopy in biopsy specimens from conjunctiva shows the accumulation of sphingolipids as PAS-positive Sudan-positive intra-lysosomal inclusions that are birefringent when viewed under polarized light [63].
- Hence, ophthalmologists often have the opportunity to identify patients early, before the disease is well advanced, emphasizing the likelihood of a heightened awareness of FD among ophthalmologists and optometrists to greatly reduce diagnostic delays and thus reduce the morbidity and mortality of this life-threatening disease [114, 115].

### Dermatological involvement

**Fig. 8:**
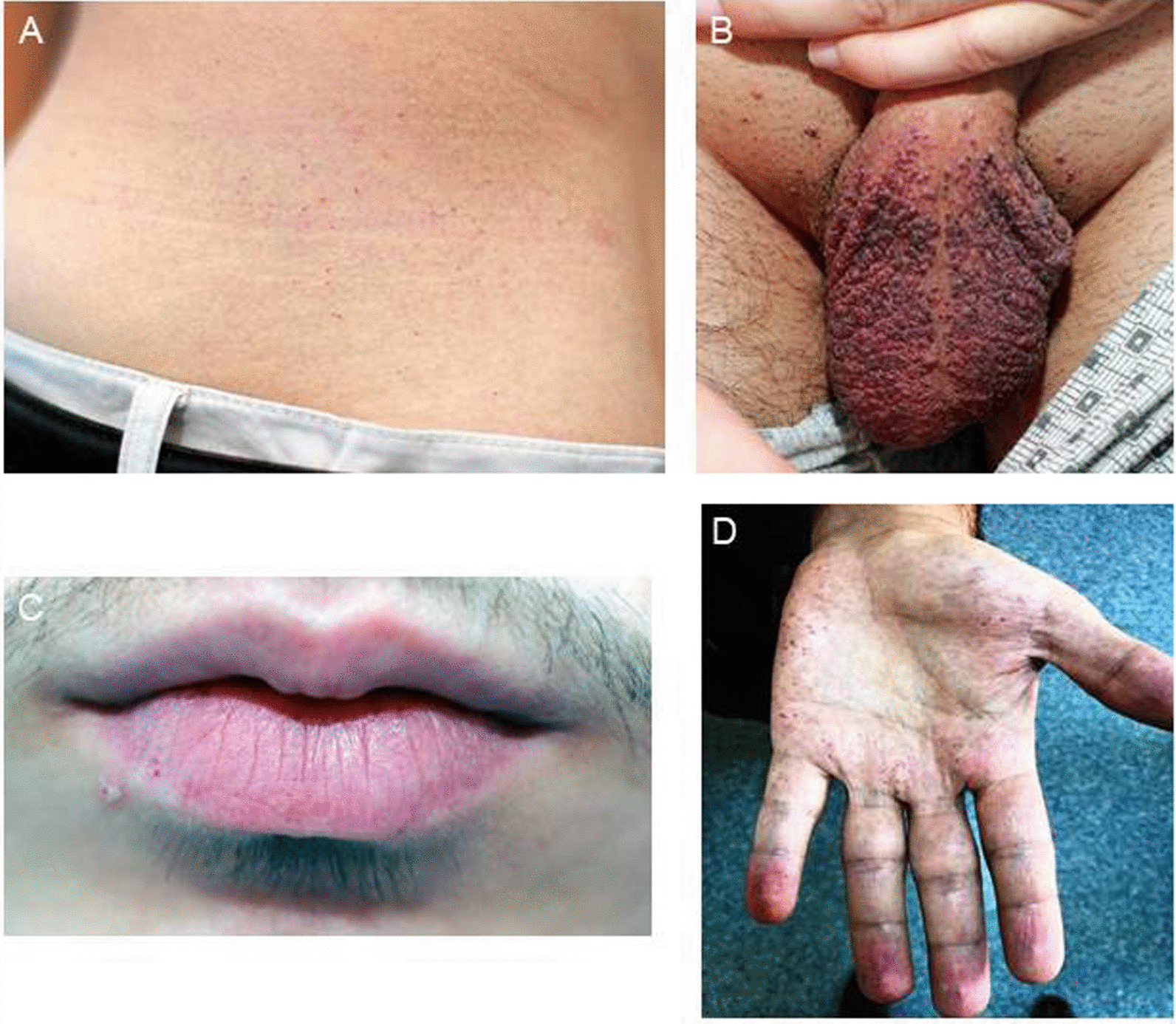
Angiokeratoma **A** multiple small pink to dark-red spots in lateral trunk, **B** multiple 1–5 mm pink to dark-red or blue-black spots with hyperkeratotic surface in scrotum, **C** spots in the lower lip, **D** spots in the palm

Dermatological abnormalities have been reported in 78% of males and 50% of females with the classic phenotype of FD [89]. Angiokeratoma is the most common dermatological abnormality occurring in 66% of males and in 36% of females, and is the most visible early clinical feature of classic FD [36]. They typically appear as clusters of small, pinkish, dark red, blue-black, non-blanching macules or papules 1–5 mm in size on the umbilicus, hands, knees, elbows, trunk and also sometimes on mucosal areas such as the mouth; spreading to the genitals during adolescence and increase in number and size with age (Fig. 8) [36, 89, 125].

Angiokeratomas frequently appear in 5–15 years of age among males and in 8–25 years of age among females [126]. They typically are higher in number and frequently located in the genital region in males, while upper back and chest regions were more common locations in females [125].

**Fig. 9:**
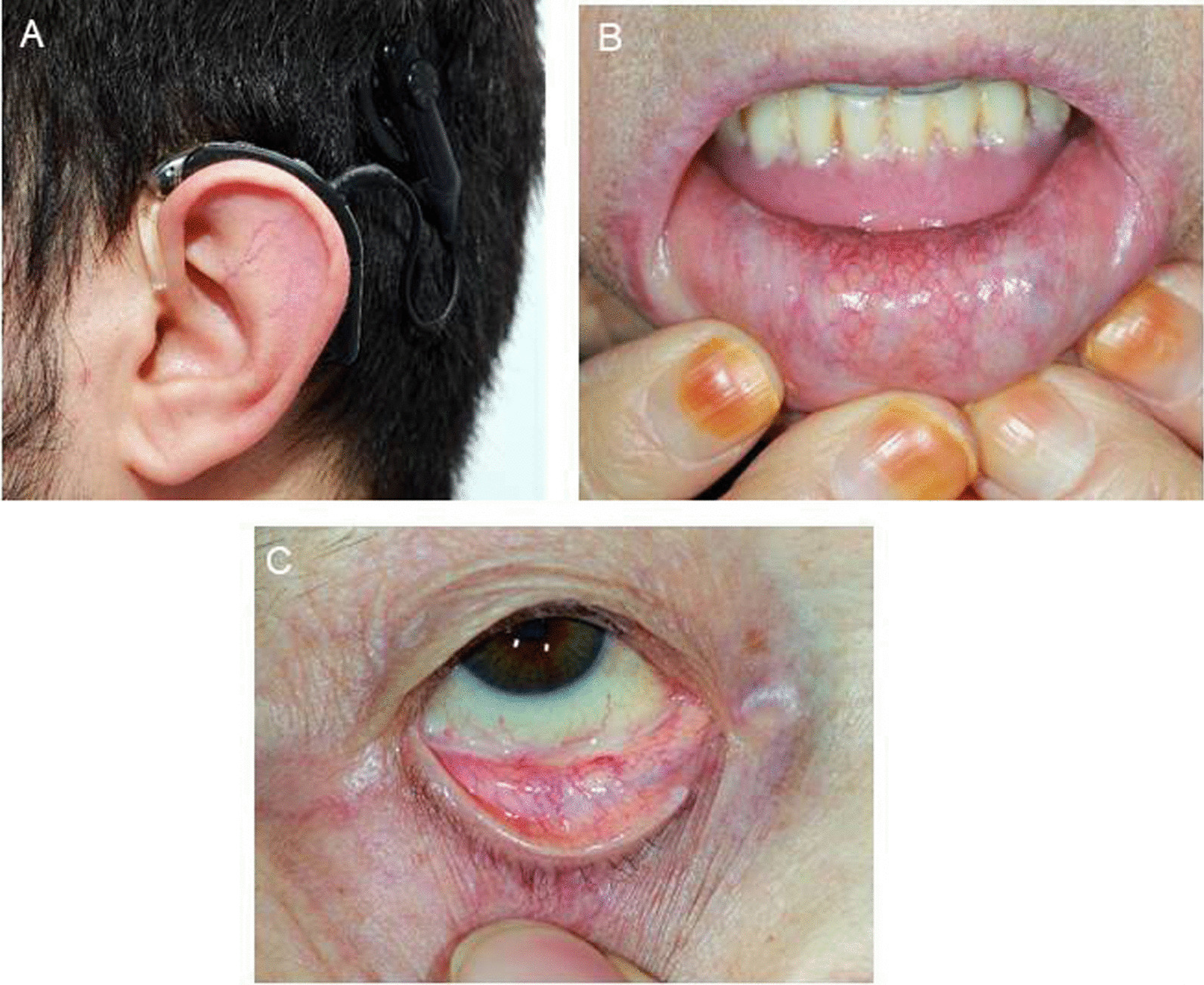
Linear telangiectasia A in the auricle in a patient with sensorineural hearing loss, **B** in the lower lip mucosa, **C** in the conjunctiva

Telangiectasia, the second-most common dermatological sign in FD, is commonly seen in sun-exposed areas such as “V” regions of the neck and face, while lip and oral mucosa are also likely locations (Fig. 9) [89, 127]. Sweating abnormalities (hypohidrosis in particular), lymphedema and hypotrichosis are other dermatological signs of FD, while facial dysmorphism may also be evident among males (peri-orbital fullness, prominent supra-orbital ridges, large bitemporal width, bushy eyebrows, broad nasal base, fullness of the cheeks and a larger chin), albeit not a prominent sign in FD unlike to other several lysosomal storage disorders [128].

#### The consensus statement on Fabry-specific dermatological involvement

- Histologically, the skin lesions are small superficial angiomas caused by cumulative damage of the vascular endothelial cells of the skin with vessel dilatation in the dermis that increase in number and size with age and can occur singly or in groups [89, 128, 129].
- Although skin biopsy may be a useful additional diagnostic test when carefully interpreted by an expert pathologist, the skin biopsies are often normal in heterozygous females and therefore not of great utility [1, 63, 130].
- Angiokeratoma may be the first sign of FD as can be seen in at least half of overall cases and in at least two thirds of male patients [80].
- Although diffuse angiokeratomas (angiokeratoma corporis diffusum) are most commonly seen in FD, they are not FD-specific lesions and can also occur in other lysosomal storage disorders (i.e. mannosidosis, fucosidosis, sialidosis, b-galactosidase deficiency and Schindler disease) as well as in the absence of any metabolic disease or enzyme defect. Nonetheless, FD should be considered in a patient when angiokeratoma corporis diffusum is accompanied with other dermatological signs such as linear telangiectasia and sweating changes (i.e. hypohidrosis) [10, 36].

## Diagnosis of FD

Despite presenting with peculiar signs and symptoms beginning in childhood, delays in diagnosis of FD are unfortunately very common with a mean diagnostic delay of 10–20 years from symptom onset to definite diagnosis [1, 6–8, 114]. Alongside the fact that FD is generally poorly understood by physicians due to their lack of knowledge about this disorder, due to a highly heterogeneous nature of the disorder clinically with a wide variety of clinical manifestations between individual carriers of the same pathogenic variants and even within the same family, the timely diagnosis of FD remains a challenge [7, 114, 131, 132]. On average, a patient with FD sees 10 specialists before the correct diagnosis is finally made and the disease usually is not diagnosed until patients are well into adulthood with average age of diagnosis of 29 years [7, 114, 131, 132].

The histopathology of kidney biopsy also provides significant evidence for FD especially when the enzyme level is uninformative, and the genetic testing reveals a VUS. The most characteristic finding on routine light microscopy of kidney biopsies is vacuolation of podocytes, of parietal epithelial cells of Bowman’s capsule, and of Henle’s loop and distal tubular cells. Mesangial widening, focal segmental glomerular sclerosis and global sclerosis, tubular atrophy, interstitial fibrosis and other nonspecific lesions are additionally seen, even at the early stages of Fabry nephropathy. On electron microscopy, the largest inclusions are seen in podocytes and in cells of the proximal and distal tubules, and Henle’s loop. In these tubular segments, affected cells can be strikingly enlarged with giant inclusions measuring up 10 µm in diameter [133].

### The consensus statement on diagnostic work-up for FD

**Fig. 10:**
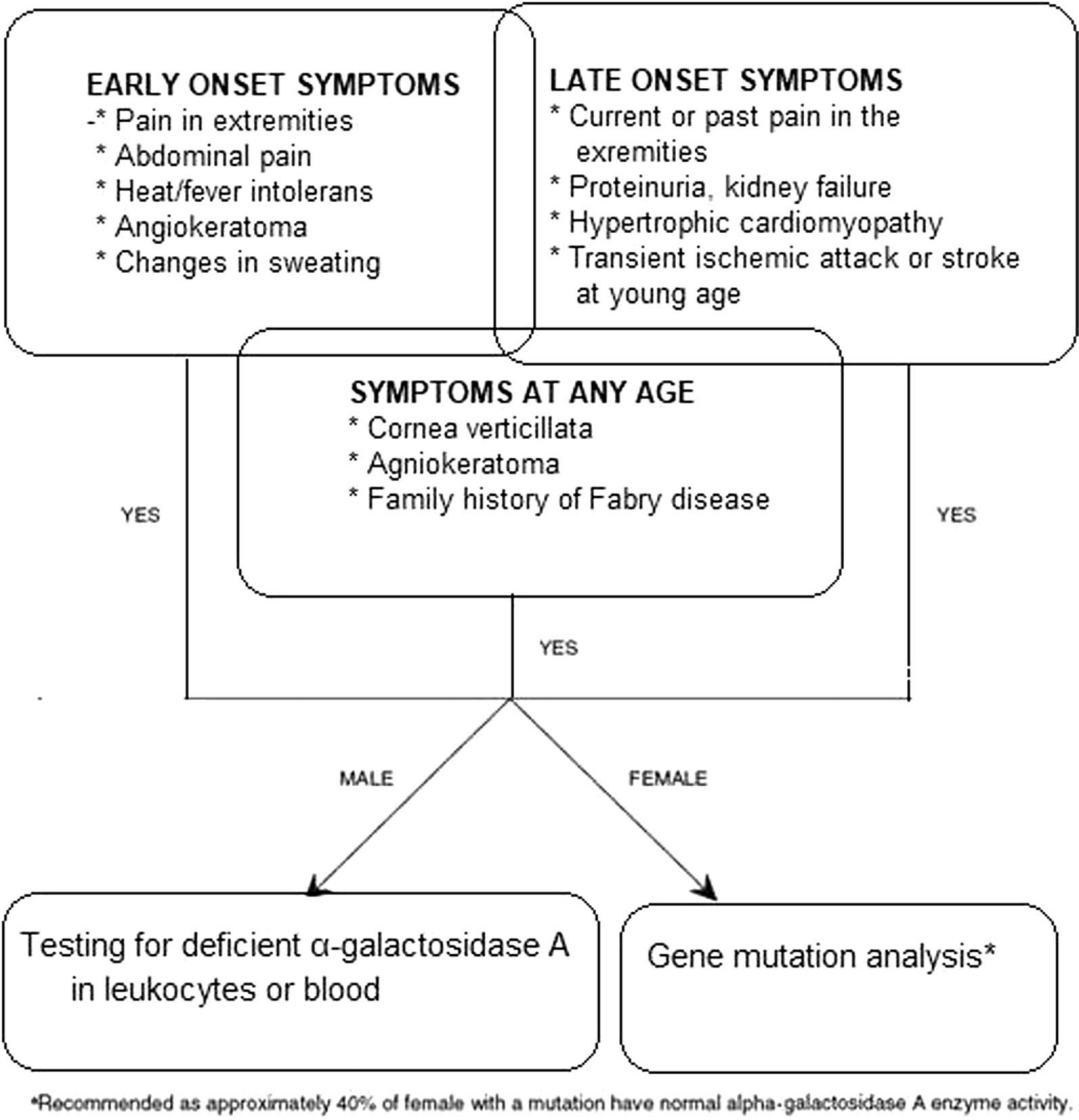
Decision-making algorithm in diagnosis of Fabry disease. Adapted from Lidove et al. Clin Genet 2012: 81: 571–577

The diagnosis of FD is based on the disease’s clinical manifestations and can be confirmed by the enzyme activity measurement, identification of glycosphingolipid accumulation, and genetic pathogenic variants studies [8]. The clinical suspicion of FD begins with identification of characteristic signs and symptoms such as neuropathic pain, angiokeratomas, ophthalmologic opacities (i.e. cornea verticillata and cataracts), anhidrosis or hypohidrosis, intolerance to exercise, heat or cold, gastrointestinal disturbances (i.e. bloating, diarrhea, abdominal pain), unexplained HCM (in men aged > 30 years and women aged > 40 years), ESRD at a young age or stroke in patients younger than 50 years with no cardio-vascular risk factors along with family history of FD and families with high prevalence of kidney disease, cardiomyopathy, or ischemic encephalopathy (Tables 2, 3, Fig. 10) [1, 4, 7, 10, 134–136].

**Table 2:** Clinical manifestations suggestive for Fabry diagnosis [1, 4, 7, 10, 134–136]

Family history of FD
Ophthalmologic deposits, such as cornea verticillata and cataracts
Anhidrosis or hypohidrosis
Angiokeratomas
Acroparesthesia (chronic neuropathic pain and episodic severe pain crises in distal extremities)
Gastrointestinal disturbances (bloating, diarrhea, abdominal pain)
Unexplained hypertrophic cardiomyopathy > 30 years for men and > 40 years for women
Personal or family history of renal failure with no cardio-vascular risk factors
End-stage renal disease at a young age
Cryptogenic stroke in patients younger than 50 years
Personal or family history of intolerance to exercise, heat or cold

**Table 3:** Clinical manifestations suggestive for common misdiagnoses [1, 4, 7, 10, 134–136]

Medical specialty	Misdiagnosis	Fabry symptom/sign
Neurology	Multiple sclerosis	Stroke
	Chronic small fiber neuropathy	Pain, tingling in hands and feet
	Raynaud syndrome	Pain/ abnormal thermal sensitivity in extremities
	Neurosis/malingering	Unexplained acute pain episodes
Rheumatology	Rheumatoid or juvenile arthritis	Joint pain, increased erythrocyte sedimentation rate
	Rheumatic fever	Pain accompanied by fever and increased ESR
	Autoimmune disorder/lupus	Angiokeratomas
	Growing pains	Unexplained pain in limbs
Nephrology	(Glomerulo) nephritis	Renal insufficiency
Cardiology	Carditis	Mitral murmur
	Hypertrophic cardiomyopathy	Hypertrophic cardiomyopathy
Dermatology	Petechiae	Angiokeratoma
Internal medicine/gastroenterology	Vasculitis	Microvascular disease
	Inflammatory bowel disease	Diarrhea, abdominal discomfort, N/V
	Appendicitis	Severe abdominal pain in the right iliac fossa
	Renal colic	Severe abdominal pain

ESR: Erythrocyte sedimentation rate; N/V: Nausea/vomiting

After diagnostic suspicion of FD is raised, the confirmation is made using laboratory testing. Detecting the deficiency in α-Gal A activity in leukocytes stands as the gold standard for the diagnosis in males. Enzyme assay using dried blood spot (DBS) is generally used for screening and a positive result or a negative result in a highly suspected patient should always be confirmed by a second tier test such as α-Gal A activity in leukocytes or genetic test. The enzyme levels in classical males is generally below 1% of normal whereas the levels may vary between 1 and 20% in late onset patients [7, 8, 39, 85]. Nonetheless, while α-Gal A activity testing alone is diagnostic for male patients; confirmation of the disease-causing *GLA* pathogenic variants is needed to establish the disease phenotype, while pathogenic variants testing is also indicated to rule out benign polymorphisms, to perform preimplantation genetic diagnosis and to permit the testing of at-risk family members [7, 8, 39, 85].

In females, α-Gal A activity can be normal due to random X-chromosomal inactivation, diagnosis in suspected cases should therefore be confirmed by demonstration of the presence of a disease-causing pathogenic variants in the *GLA* gene via genetic analysis [4, 5, 7, 8, 39, 85].

Accordingly, given the role of molecular analysis of the *GLA* gene as an essential diagnostic or prognostic tool providing data on the exact kind of variants and related course of clinical manifestations, awareness of the treating physician about the different kinds of pathogenic variants and their clinical implications is highly important [7, 137].

Histopathologic examination of the kidney biopsy samples is of significant importance to provide evidence for FD when genetic testing reveals new variants (especially VUS).

FD is a multi-systemic disease with diverse range of possible alternative diagnoses related to several medical specialties, clinicians should therefore consider a diagnosis of FD when dealing with a wide range of symptoms that may be either specific to FD (both early- and late-onset symptoms) or non-specific, but which could fit within the phenotype of FD to address the diagnostic delay in FD and facilitate earlier therapy, leading to better outcomes [10].

Considering the significant number of applications to outpatient clinics and the increased risk of losing for follow-up in Turkey, enzyme analysis and genetic testing should be ordered simultaneously in male patients, whereas genetic testing should be the first option for diagnosing females as it is widely available in Turkey.

## Biomarkers for FD

There is no ideal biomarker in FD, while microalbuminuria, proteinuria, and serum creatinine are the usual biomarkers for renal monitoring. Nonetheless, some rarely used biomarkers such as cystatin C, beta 2-microglobulin (β2M) and neutrophil gelatinase-associated lipocalin/lipocalin-2 creatinine have also been investigated in FD patients [138]. Hence, authors noted that along with serum creatinine, measurement of β2M or cystatin C should be considered for renal evaluation of FD patients [138]. Similarly, the utility of the amino-terminal fragment of the brain natriuretic propeptide (NT-proBNP) has subsequently been confirmed as a diagnostic and prognostic predictor of heart disease [139, 140].

Plasma Lyso-Gb3 levels, deacylated form of Gb3 rather than urine or plasma Gb3levels are considered more sensitive markers. Although in some studies correlation of lyso-Gb3 levels with some cardiac and neurological manifestations other studies have not found such associations [6, 141]. Lyso-Gb3 contributes to the pathology of disease [1, 42, 142–144]. Nonetheless, the utility of LysoGb3 is still controversial, due to concerns that LysoGb3 levels may not be strongly associated with disease phenotype [13]. Other potential biomarkers in FD include plasma 3-nitrotyrosine, podocyturia and urinary excretion of CD80 [145, 146], while sphingosine-1-phosphate (S1P) was recently identified as a biologically active growth-promoting factor involved in cardiovascular remodeling in both males and females with FD plasma levels of which show a strong correlation with LVM index, and increased common carotid artery intima-media thickness [147]. There is a need for studies to help identify new therapeutic targets and reliable biomarkers for diagnosis and prognosis of FD as well as to assess treatment response [1, 139].

## Clinical suspicion, over suspicion, screening and genetic counselling

Screening individuals with a family history of FD or newborn screening programs are the only practical ways of identifying patients before the development of symptoms, while screening of patients in high risk groups who may be exhibiting late-onset symptoms of FD is also considered important in optimizing the management of disease in these patients [1].

Once the diagnosis has been confirmed, the opinion of a pediatric metabolic physician, pediatric or medical geneticist should be sought and family screening carried out, and the Pedigree analysis and effective screening of the family of a newly diagnosed patient is likely to result in identification of several previously unrecognized affected family members, including young relatives at a relatively early stage of their disease [148, 149].

The high-risk populations include individuals with LVH, ESRD and crytogenic stroke and/or small-fiber neuropathy [36] as reported to be associated with 1.14%, 0.3% and 1.6% of FD prevalence, respectively in a systematic review of previous large studies [150]. In fact, screening studies for FD may reveal individuals with genetic variants of yet unknown significance and may inadvertently indicate falsely higher prevalence due to benign polymorphisms (e.g., D313Y) [23, 39, 150].

### The consensus statement on interpretation of pathogenic variants

**Fig. 11:**
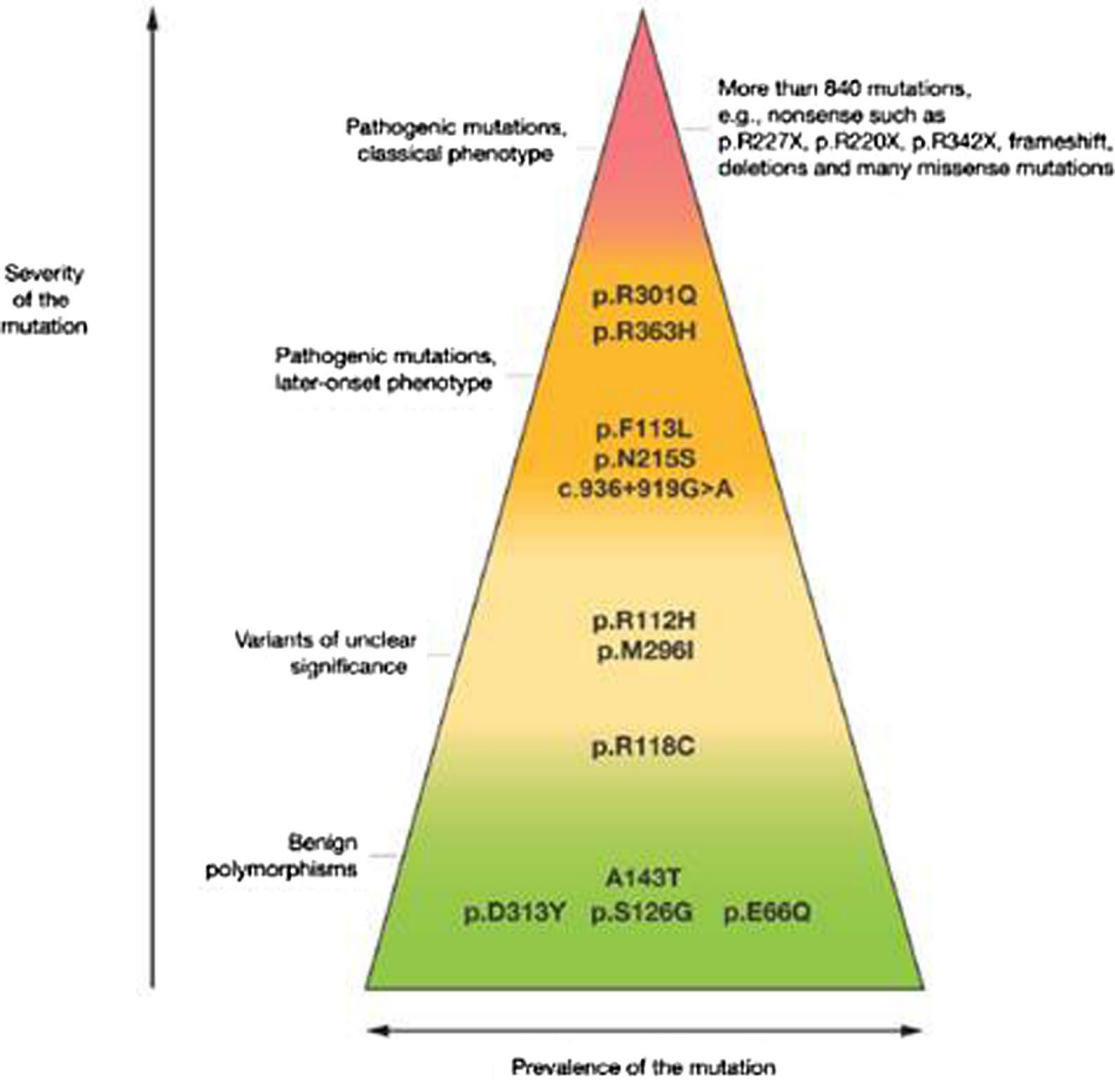
Some key mutations associated with the classic or later-onset Fabry disease phenotype, GLA variants of unclear significance (VUS), and benign variants. The triangular form illustrates the higher frequency of benign and probably benign variants. Physicians should be aware that, due to this higher frequency, such mutations may be seen in screening studies but may not be related to actual Fabry-related manifestations. Adapted from Ortiz et al. Mol Genet Metab. 2018 Apr; 123(4):416–427

Nowadays, FD diagnosis rate is increasing via pedigree analysis or family screening after diagnosis of another family member, and use of DNA sequencing technologies (either Sanger sequencing or Next Generation Sequencing) rather than enzymatic screening is becoming more widespread for screening of at-risk groups. However, while is a useful innovative technology, it may lead to the identification of genetic variants of unknown significance (VUS), which are often not associated with the characteristic features of FD [1, 4, 8]. This seems notable given the challenges in interpretation of genetic *GLA* pathogenic variants to predict the pathogenicity for a *GLA* VUS, which necessitates further clinical, biochemical, or histopathological evidence of FD in individuals with a *GLA* VUS to determine the pathogenic nature of the pathogenic variants, before initiation of therapy [1, 4, 39, 151]. In this regard, de novo pathogenic variants which were not described before require further clinical investigation including brain and cardiac MRI, an electron microscopy analysis of the biopsy of the affected organ to look for Gb3 deposits and a thorough family testing to find and investigate males with low α-Gal A activity and the same pathogenic variants that would support variants being pathogenic [7, 12, 39, 136, 152, 153]. In addition, most of the pathogenic *GLA* pathogenic variants are private, occurring in a single or few families; intra-familial phenotypic variability has been observed, complicating the study of genotype–phenotype correlations (Fig. 11) [1, 39].

Accordingly, while the identification of pathogenic variants within the *GLA* gene is important to the diagnosis of FD, other questions have emerged regarding how to elucidate VUS and how to explore potential genotype–phenotype relationships to perform correct risk stratification [8, 13] While the “gold standard” to clarify if a novel pathogenic variant is likely pathogenic or likely benign includes in vitro *GLA* pathogenic variants expression assays, this is only available at specialized research laboratories [148]. There are also controversial variants [7], like the p.D313Y and some intronic variants, described early in literature as pathogenic by some authors [154] and later as benign by others [155]. Therefore, the advice of an expert in pediatric metabolic diseases, as well as pediatric or medical genetics should be sought for interpretation of the pathogenicity of any VUS [39], while more importantly, the treating physician should always rely on the patients’ clinical manifestations and examination, besides the genotype, when deciding whether to begin treatment [7, 156].

There is increasing awareness of FD among primary care physicians and different specialists, and systematic screening among high-risk populations and newborns has become more frequent [13]. Notably, a new emergence challenge in the field of Fabry diagnosis is over suspicion due to increased awareness of a rare disease that has become treatable with ERT and thus the idea of having a specific therapy to offer to a potential patient made the physician to put FD as one of the first in the list of diseases for differential diagnosis when only one sign of symptom is present in his or her patient [12]. This has been associated with remarkable increase in the number of samples received by reference labs specialized in Fabry diagnosis and thus decline in the proportion of positive cases along with increased detection of pathogenic variants with unknown clinical relevance [13, 136, 157]. This emphasizes the need for a more complete clinical picture, medical history, and family history in order to decide, especially in females, which samples should be tested [12].

Accordingly, screening based on solid criteria for the high clinical suspicion and correct interpretation of pathogenicity of pathogenic variants (i.e., awareness of polymorphisms like p.D313Y p.E66Q, and probably p.R118C with high residual activity) are crucial to establish final diagnosis [12, 39].

Based on the reports about the pathogenic variant spectrum of the patients from Turkey, consistent with the rest of the world, recurrent pathogenic variants are rare [14, 158].

It is a fact that enzyme assay from dried blood spots could reveal false positive or negative results unless confirmed by a second-tier test. The novel variants in the *GLA* gene could be misinterpreted as “pathogenic” due to various reasons. Based on these facts it is highly possible that some cases have erroneously been put on therapy although they do not have Fabry Disease [7].

## Treatment of FD

**Fig. 12:**
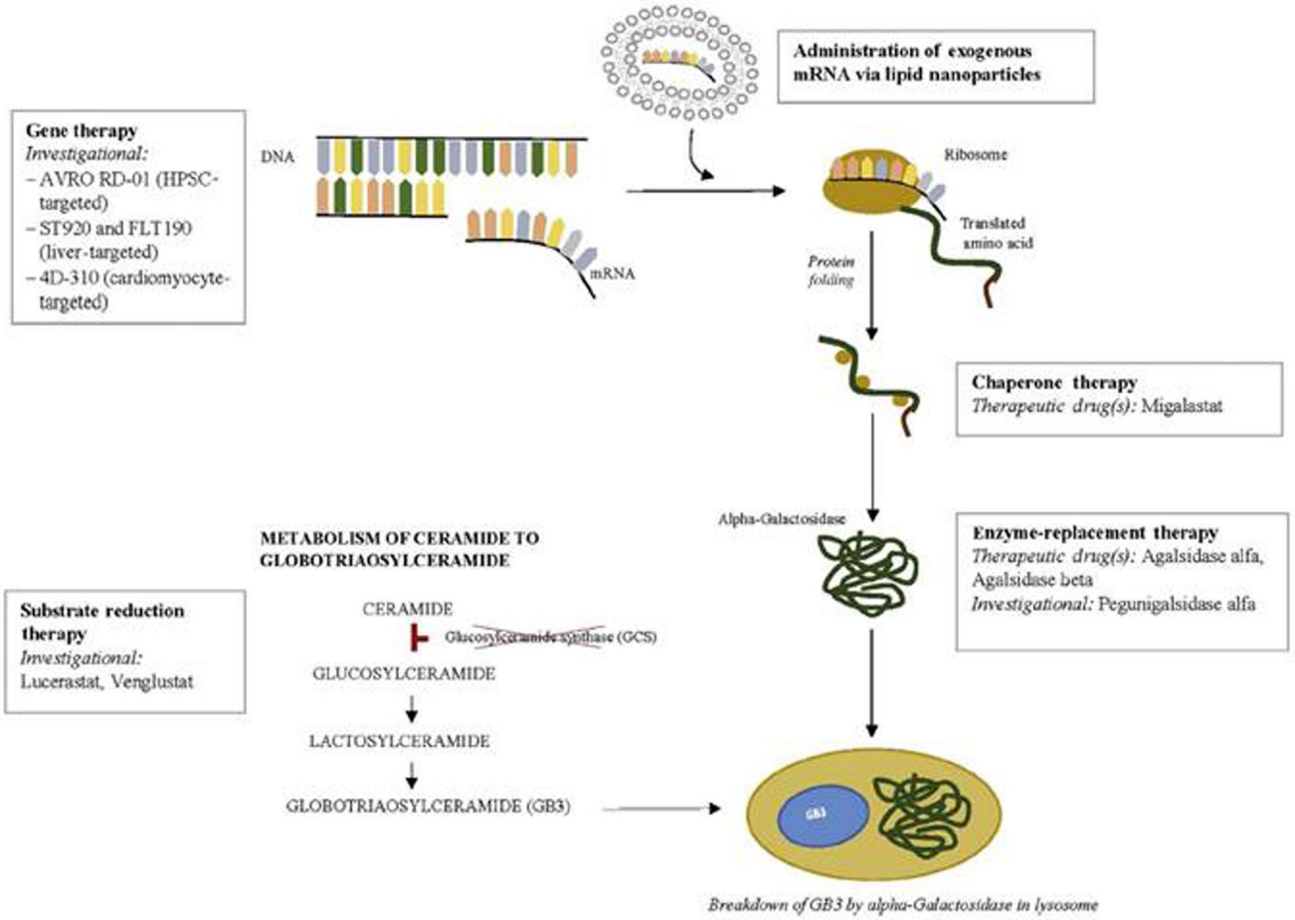
Current and investigational therapeutic agents for Fabry disease. Adapted from Felis et al. Kidney Int Rep. 2019 Dec 6; 5(4):407–413

FD is characterized by progressive, multi-organ pathology manifesting as a range of clinical phenotypes affecting both genders in terms of poor QoL, morbidity and shortened life expectancy [1, 36]. Currently, ERT and chaperone therapy are the two therapeutic modalities available clinically for the treatment of FD, while other alternatives such as substrate reduction therapy, mRNA based therapy, and gene therapy are in development (Fig. 12) [42, 159].

ERT, the first approved disease-specific therapeutic option for patients with FD, remains the mainstay of treatment for most patients as associated with improved patient QoL and stabilized kidney function worsening [159]. However, given the unmet clinical needs via ERT, investigational therapies are directed at either replacing or generating deficient enzyme, or blocking the accumulation of substrate [159]. Strategies targeting enzyme delivery or production include modification of the enzyme to increase the duration of therapeutic plasma concentrations, mRNA administration, and gene therapy, while non-enzyme-replacement strategies including substrate reduction therapy are currently in clinical trials and aims to reduce GB-3 production by inhibiting glucosylceramide synthase [159]. Chaperone therapy, recently approved (although not available in Turkey yet), stabilizes the endogenous enzyme in patients with amenable pathogenic variants to increase enzyme activity (Fig. 12) [159].

### Enzyme replacement therapy

For ERT, there are two available pharmaceutical preparations of recombinant human α-Gal A including agalsidase alfa (Replagal® by Shire Human Genetic Therapies, Lexington, MA) and agalsidase beta (Fabrazyme® by Sanofi Genzyme, Cambridge, MA) produced in a lineage of human fibroblasts and in Chinese Hamster Ovary (CHO) cells, respectively. Both preparations have similar glycosylation patterns, specific activities, and enzyme kinetics [160] and both have been shown to be clinically efficacious [9, 32]. Both preparations are administered intravenously every other week and indicated for long-term treatment [36, 42]. Agalsidase alfa is given with a dose of 0.2 mg/kg and agalsidase beta is given as 1 mg/kg both with biweekly infusions [17, 37, 161, 162].

Clinical trials, observational studies and registry data have provided many evidences for safety and efficacy of ERT in improving symptoms of pain, gastrointestinal disturbances, hypohidrosis, left ventricular mass index, GFR and QoL [98, 163]. ERT is considered able to stabilize the pathogenic processes, prevent progression of the disease, improve disease outcome, and increase the QoL among FD patients [8, 164]. For the renal phenotype, ERT seems to stabilize kidney function worsening in these patients and at least slow their decline in renal GFR [58]. For the cardiac phenotype, ERT is able to stabilize or improve surrogate parameters like cardiac size among individuals with cardiomyopathy [165]. Data published in adult male patients with FD demonstrates that the efficacy of ERT with consistent, dose-dependent reductions in Gb3 accumulation, a reduced decline in eGFR, and improvements in cardiac outcomes along with gastrointestinal, pain and QoL outcomes or stabilization of a lifetime progressive debilitating disease [37].

Nonetheless, ERT has some limitations due to a restricted volume of distribution, requirement for intravenous access, and stimulation of the production of anti-drug antibodies as well as limited efficacy including continued progression of cardiac fibrosis and progression of white matter disease on ERT [159, 166]. Pegunigalsidase alpha is a novel pegylated form of α-GAL produced in a PlantCell Ex system and an investigational ERT with preclinical data supporting longer circulatory half-life, increased cardiac and renal uptake and decreased hepatic uptake than currently available ERT preparations [167, 168]. The data from an open-label, 3-month pharmacokinetics study followed by 9 months of follow-up also confirmed a longer half-life (average 80 h) when compared to half-life of 2 h for existing therapies along with a favorable tolerability profile and a 50% reduction in GB-3 in majority of patients, particularly in those with classic pathogenic variants [169].

In Turkey, generally the criteria for initiating ERT for patients is parallel to the international recommendations stating that symptomatic patients as well as asymptomatic boys with classical Fabry pathogenic variants around age 8–10 years should be considered for treatment [39, 170].

### Chaperone therapy

Some missense mutations within the *GLA* gene often result in an unstable and misfolded protein, leading to reduced intracellular AGAL activities. Misfolded proteins will not pass the protein quality-control mechanism within the endoplasmic reticulum (ER), resulting in premature degradation before reaching the lysosomes. To restore folding and stability of the protein, pharmacological chaperones can be used, binding reversibly to the active center of the protein [171]. Migalastat (Galafold; Amicus Therapeutics, Cranbury, NJ), a pharmacological chaperone, is an orally bioavailable iminosugar that increases available enzyme activity in patients with pathogenic variants s amenable to the therapy [159, 166]. It has been associated with improved protein folding and promoted trafficking of the protein to the lysosome by binding to the defective *α-GAL* in the endoplasmic reticulum [172]. Two phase III studies were conducted with migalastat conducted. The FACETS study is a placebo-controlled study including treatment-naïve patients (N = 50; 22 placebo vs. 28 migalastat). Although there was no significant difference between the number of responders (≥ 50% reduction in the number of GL-3 inclusions per kidney interstitial capillary) in the placebo and migalastat groups, six months of migalastat was associated with a significantly greater reduction in the mean (± SE) number of GL-3 inclusions per kidney interstitial capillary than was placebo. In patients who received migalastat for up to 24 months, a significant decrease in the left-ventricular-mass index was observed [173]. In the ATTRACT study, migalastat is compared to enzyme replacement (agalsidase-beta, agalsidase-alfa) in 57 patients (36 migalastat vs. 21 ERT males and females). Renal function was stable for 18-month treatment period with migalastat, a signifcant reduction of the left ventricular mass index was observed, and plasma lyso-Gb3, as a marker of disease burden, remained low and stable when switching from ERT to migalastat [174].

Recently, a single-center observational study on seven male Fabry patients (18–66 years) who switched from ERT to migalastat treatment revealed that cardiac, renal and neurologic functions, and FD-related symptoms and questionnaires were stable between baseline and the switch, and remained unchanged with migalastat. A significant improvement was observed in left ventricular mass index from baseline (diagnosis of FD) to T2 (1 year of therapy with migalastat) (*p* = 0.016), with a significative difference between the treatments (*p* = 0.028), and in median proteinuria from T2 versus T1 (*p* = 0.048) [175].

There are more than 1000 mutations in the GLA gene known to be associated with Fabry disease; an estimated 35–50% of patients with Fabry disease have mutations that are amenable to migalastat therapy. Based on a HEK 293 cell based assay, migalastat-amenable mutations are defined as those in GLA that translate to mutant forms of α-galactosidase A and display a ≥ 1.2-fold increase in α-galactosidase A activity over baseline and an absolute increase of ≥ 3% over wild-type α-galactosidase A activity, in the presence of 10 μmol/L migalastat [176].

#### Substrate inhibition therapy

Substrate reduction therapies such as lucerastat (Idorsia Pharmaceutical Ltd, Allschwil, Switzerland) and venglustat (Sanofi Genzyme, Cambridge, MA) function as a glucosylceramide synthase inhibitor preventing accumulation of GB-3 by limiting the amount of ceramide that is converted to glycosphingolipid [177]. Preliminary data from clinical trials evaluating the effect of Venglustat in treatment-naïve Fabry patients suggest a slow but gradual clearance of Gb3 from superficial skin capillary endothelium and a gradual decrease of plasma lysoGb3 in most included patients over the course of 3 years of treatment [178].

### Gene therapy

Gene therapies include gene editing with the ex vivo approach where hematopoietic stem cells harvested from the patient are infused back into the patient after gene editing and the in vivo approach where a vector with gene editing is infused directly into the patient, and then cells within the patient, such as liver cells, directly undergo gene editing to express the missing protein [160].

Administration of *α-GAL* mRNA to stimulate production of *α-GAL* without the need for either myeloablative therapy or administration of viral vectors for gene transduction is another therapeutic approach that is currently under testing [160]. Nonetheless, whether or not the current gene therapy approaches will achieve stable viral copy numbers and sufficient α-GAL A activity compared to currently available or developing long-lived ERT preparations remains to be clarified for gene therapy approaches to be adopted as therapeutic interventions for FD [159, 179].

### Adjunctive therapies

Treatment with ERT should be combined with supportive interventions, if indicated, to clinically manage the renal, cardiac, neurological, and other complications of Fabry disease-induced chronic tissue injury [39].

Adjunctive treatment includes analgesic drugs, reno-cardio-protection [angiotensin converting enzyme (ACE) inhibitors and angiotensin receptors blockers (ARBs), statin therapy, adherence to a low-sodium diet, antiarrhythmic and anticoagulant agents and vitamin D repletion as needed] and lifestyle modifications (e.g., avoiding extremes of temperature), whereas dialysis or renal transplantation are available for patients experiencing ESRD (Table 4) [1, 23, 39, 180].

**Table 4:** Adjunctive support for the management of Fabry disease [39, 180]

Organ/system involvement	Adjunctive/symptomatic therapy and preventative measures
*Renal*	
Proteinuria and reno-protection	ACE inhibitor or ARB
	Dietary salt restriction
	Statin therapy
	Vitamin D replacement therapy if needed
Renal failure	Dialysis or kidney transplantation (donor screened negative for FD)
*Cardiac*	
Hypertension	ACEI or ARB (beta blockers should be used with caution and amiodarone avoided in patients receiving ERTa)
Symptomatic bradycardia/chronotropic incompetence or significant AV conduction impairment	Permanent cardiac pacing
Atrial fibrillation	Lifetime anticoagulation with maintenance of sinus rhythm
Malignant arrhythmias	Implantable cardioverter-defibrillator
*Neurologic*	
Stroke prophylaxis	Antithrombotic agents (aspirin or clopidogrel) as secondary prevention; no data available regarding primary prevention
	Anticoagulants (warfarin or the new anticoagulant drugs in absence of kidney failure), when needed, e.g., patients with atrial fibrillation
Neuropathic pain management	First-line agents include anticonvulsants (e.g., carbamazepine, gabapentin, pregabalin); other drugs can be considered according to current international recommendations for neuropathic pain
Pain crises	Opioid agonists (care needed to avoid worsening GI disturbances)
Avoiding pain triggers	Lifestyle modifications (e.g., avoid temperature extremes, maintain proper hydration, use air conditioning, cooling vests, facial mist/spray)
*Psychiatric*	
Depression, anxiety	Psychiatric referral, pain center referral, serotonin reuptake inhibitors
*Gastrointestinal*	
Delayed gastric emptying	Metoclopramide
Dyspepsia	H-2 blockers
Dysmotility and diarrhea	Dietary changes (increased fiber intake, more frequent and smaller meals) and pharmacotherapy
*Pulmonary*	
Airway obstruction	Bronchodilators
*Ophthalmological*	
Difficulty in driving at night	Polarized glasses
	Artificial tears ointment
*ENT*	
Hearing loss	Hearing aids, cochlear implants
Vertigo-related nausea	Trimethobenzamide, prochlorperazine
*Dermatological*	
Angiokeratomas	Laser/cosmetic treatment (not proven effective)
Lymphedema	Compression stockings

ACE: Angiotensin converting enzyme; ARB: Angiotensin receptor blocker; AV: Atrioventricular; ENT: Ear-nose-throat; ERT: Enzyme replacement therapy; FD: Fabry disease

#### The consensus statements on management of FD

- The general therapeutic goal for optimizing patient management in FD should be to optimize both disease-specific and nonspecific adjunctive treatments to prevent or minimize effects of organ damage (e.g. kidney dysfunction) and prevent clinical events (e.g. stroke) as well as reduce symptoms, such as neuropathic pain [36].
- The management of FD should be carried out by an experienced multidisciplinary team based on an individualized approach to patient care consistent with the natural history of the specific disease phenotype and should involve early ERT initiation after comprehensive evaluation of disease involvement, routine monitoring for evidence of organ involvement in late onset asymptomatic patients and response to therapy in treated patients to regularly re-evaluate and appraise the therapeutic goals and use of adjuvant treatments to assist in the management of organ-specific complications [36, 39].
- There is growing evidence that early initiation of ERT optimizes treatment benefits, and can potentially prevent or delay progression to permanent organ damage [36]. Accordingly, the present expert panel agreed that ERT should be initiated as soon as possible on presentation of early clinical signs related to kidney, heart, or brain involvement in order to achieve the best efficacy and to avoid irreversible pathological changes given that the earlier treatment with ERT is begun, the greater the potential for benefit [1, 8, 85, 181].
- For male patients with a classic Fabry pathogenic variants, ERT should be initiated promptly when there are clinical manifestations, but should be considered for males older than 10 years of age even in the absence of clinical signs or symptoms of organ involvement. In addition, female patients with classical pathogenic variants or patients with later onset Fabry pathogenic variants should be treated as soon as possible ERT if they present with symptoms suggesting major organ (kidney, heart, or brain) involvement or laboratory, histological, or imaging evidence of injury to the major organs.
- Tissue-based assessment of FD pathology may assist the decision to initiate ERT and may occasionally be helpful in assessing disease progression and response to treatment during follow-up. The treatment should not be withheld from patients with severe renal insufficiency or with renal transplantation, from those on dialysis, or those with cognitive decline [8, 39].
- Given the rising number of pregnant women with FD, it should also be noted that the agalsidase alfa [182] and agalsidase beta [183] treatments was reported to be safe and well tolerated during pregnancy and lactation, with no adverse events reported in mothers and children in the case series studies. Nevertheless, further studies in pregnant FD patients are needed to better understand the benefits and risks of therapy and to justify the universal use of ERT among all pregnant women with symptomatic FD [182, 183].
- Strategies to optimize the management FD patients involves timely use of ERT along with appropriate non-specific adjunctive therapies through an individualized optimal treatment strategy suiting patient’s clinical signs and symptoms and improving QoL which is often impaired even in early-stage disease because of neuropathic pain, whereas a need for further investigation is obvious that would address the potential invasive or non-invasive markers of disease progression as well as the impact of seroconversion and IgG antibody production on treatment response among FD patients [36].
- While the advent of potential alternative therapeutic agents hold potential promise for FD patients, their combined use should also be investigated in future studies in terms of potential to enable better outcomes and treatment of organ-specific sequelae of FD should be continued via standard-of-care adjunctive therapies [159].
- Being the only approved treatment option in Turkey, enzyme replacement therapy stays as the first choice for the etiology targeted treatment in indicated patients.

## Multidisciplinary approach for evaluation and management

Given its multi-systemic nature, setting up a medical care plan for FD should ensure interdisciplinary communication across the specialties managing different organ complications [4, 36]. Awareness in medical community about FD should be one of the main activities from reference professionals in the field of lysosomal diseases [12]. Therefore, cardiologists, neurologists, dermatologists, nephrologists and ophthalmologists should all be aware of the possibility of FD, depending on the patient’s clinical presentation [4]. Moreover, the effective management of FD requires a multidisciplinary approach and a follow-up program that involves comprehensive teamwork, which should ideally be supervised by physician experienced in the management of patients with FD, with input from sub-specialists who also have FD experience, as part of a multidisciplinary clinical team that includes pediatric metabolic physicians, pediatricians, ophthalmologists, nephrologists, cardiologists, neurologists, gastroenterologists, dermatologists, genetic counselors, pediatric or medical geneticists, psychologists, trained pathologists, and professional nurses [8, 38, 162].

## Conclusion

This consensus statement indicated the clinical heterogeneity of FD as well as a large number of pathogenic variants in the *GLA* gene, emphasizing a need for an individualized approach to patient care in accordance with genotype, gender, family history, phenotype, and specific clinical symptom severity of a given patient. The experts reached consensus on the critical role of a high index of suspicion in symptomatic patients and screening of certain at risk groups to reveal timely and accurate diagnosis of FD along with consideration of various causes of small-fiber neuropathy, cryptogenic young stroke and sarcomeric HCM in the differential diagnosis. The expert panel emphasized the need for increased awareness of the treating physician about the different kinds of pathogenic variants s and their clinical implications as well as necessity of further clinical, biochemical, or histopathological evidence of FD to determine the pathogenicity of VUS variants in *GLA* gene. The expert panel agreed on the diagnostic and prognostic role of histological changes on renal biopsy as become evident before clinical and laboratory indicators of disease, the role of MRI in differential diagnosis of Fabry-specific cardiac and cerebrovascular involvement and higher specificity of ocular manifestations with higher opportunity of ophthalmologists to identify FD patients at early period. The expert panel emphasized a need for a coordinated, multidisciplinary care approach in the management of FD with early start of treatment, tailoring the treatment to the needs of individual patients and regular assessment of disease progression in all patients to obtain the therapeutic goals for improving QoL and reducing progression of the disease or to stabilize end organ structure and function. The experts emphasized the crucial role of timely recognition of FD with minimal delay from symptom onset to definite diagnosis in better management of FD patients, given the likelihood of changing the disease’s natural history, improving the patients’ QoL and the prognosis via ERT once a diagnosis is made. In this regard, this consensus document is expected to increase awareness among physicians about unique characteristics of FD to assist clinicians in recognizing FD with a well-established clinical suspicion consistent with pathogenic variants and gender-based heterogeneous clinical manifestations of FD and in translating this information into their clinical practice for best practice in the management of patients with FD.

## Data Availability

All data are available through cited literature.

## Abbreviations

α-Gal A: α-Galactosidase A
β2M: Beta 2-microglobulin
CKD: Chronic kidney disease
CWML: Cerebral white matter lesions
ERT: Enzyme replacement therapy
ESRD: End-stage renal disease
FD: Fabry disease
Gb3: Globotriaosylceramide
GFR: Glomerular filtration rate
GI: Gastrointestinal
HCM: Hypertrophic cardiomyopathy
IBS: Irritable bowel syndrome
LVH: Left ventricular hypertrophy
MRI: Magnetic resonance imaging
MRS: MR spectroscopy
NT-proBNP: Brain natriuretic propeptide
PET: Positron emission tomography
QoL: Quality of life
TCD: Trans-cranial Doppler
TIA: Transient ischemic attack
VUS: Variants of unknown significance
